# Altered frontal connectivity as a mechanism for executive function deficits in fragile X syndrome

**DOI:** 10.1186/s13229-022-00527-0

**Published:** 2022-12-09

**Authors:** Lauren M. Schmitt, Joy Li, Rui Liu, Paul S. Horn, John A. Sweeney, Craig A. Erickson, Ernest V. Pedapati

**Affiliations:** 1 Cincinnati Children’s Hospital Medical Center, 3333 Burnet Ave, MLC 4002, Cincinnati, OH 45229 USA; 2 University of Cincinnati College of Medicine, Cincinnati, OH USA

**Keywords:** Fragile X syndrome, FXS, Electroencephalography, EEG, Connectivity, Executive function

## Abstract

**Background:**

Fragile X syndrome (FXS) is the leading inherited monogenic cause of intellectual disability and autism spectrum disorder. Executive function (EF), necessary for adaptive goal-oriented behavior and dependent on frontal lobe function, is impaired in individuals with FXS. Yet, little is known how alterations in frontal lobe neural activity is related to EF deficits in FXS.

**Methods:**

Sixty-one participants with FXS (54% males) and 71 age- and sex-matched typically-developing controls (TDC; 58% males) completed a five-minute resting state electroencephalography (EEG) protocol and a computerized battery of tests of EF, the Test of Attentional Performance for Children (KiTAP). Following source localization (minimum-norm estimate), we computed debiased weighted phase lag index (dWPLI), a phase connectivity value, for pairings between 18 nodes in frontal regions for gamma (30–55 Hz) and alpha (10.5–12.5 Hz) bands. Linear models were generated with fixed factors of group, sex, frequency, and connection. Relationships between frontal connectivity and EF variables also were examined.

**Results:**

Individuals with FXS demonstrated increased gamma band and reduced alpha band connectivity across all frontal regions and across hemispheres compared to TDC. After controlling for nonverbal IQ, increased error rates on EF tasks were associated with increased gamma band and reduced alpha band connectivity.

**Limitations:**

Frontal connectivity findings are limited to intrinsic brain activity during rest and may not generalize to frontal connectivity during EF tasks or everyday function.

**Conclusions:**

We report gamma hyper-connectivity and alpha hypo-connectivity within source-localized frontal brain regions in FXS compared to TDC during resting-state EEG. For the first time in FXS, we report significant associations between EF and altered frontal connectivity, with increased error rate relating to increased gamma band connectivity and reduced alpha band connectivity. These findings suggest increased phase connectivity within gamma band may impair EF performance, whereas greater alpha band connectivity may provide compensatory support for EF. Together, these findings provide important insight into neurophysiological mechanisms of EF deficits in FXS and provide novel targets for treatment development.

**Supplementary Information:**

The online version contains supplementary material available at 10.1186/s13229-022-00527-0.

## Background

Fragile X syndrome (FXS) is the leading monogenic inherited form of intellectual disability (ID) and autism spectrum disorder (ASD) and is caused by a cysteine-guanine-guanine (CGG) trinucleotide repeat expansion in the fragile X messenger ribonucleoprotein 1 (*FMR1*) gene (> 200 repeats) located on the X chromosome [1, 2]. The repeat trinucleotide expansion in the *FMR1* gene leads to a decrease or absence of fragile X messenger ribonucleoprotein (FMRP) expression, which is essential for brain development and cognitive function [2].

It is well documented that individuals with FXS exhibit impairments in executive functions (EF), an important set of cognitive functions involved in adaptive goal-oriented behavior, including subdomains of cognitive flexibility, working memory, response inhibition, and processing speed [3]. EF impairments are present to a lesser degree in females with FXS compared to males with FXS, as expected based on obligate mosaicism [4, 5]. However, even in the absence of general cognitive impairment in females with FXS, EF impairments are still observed [3]. Likewise, even though individuals with FXS are at a disproportionally higher risk of a comorbid attention deficit/hyperactivity disorder (ADHD) diagnosis, EF impairments are present even in the absence of ADHD. EF impairments can cause significant distress to both the individual with FXS and their families and is a common reason for clinic visits [6–8]. Yet, our lack of understanding of its underlying physiology has stalled treatment development targeting EF for individuals with FXS despite its critical clinical significance.

EF historically has been associated with frontal lobe function with distinct domains of EF demonstrating a degree of regional specificity within the frontal lobe [9]. Although few structural or functional magnetic resonance imaging (MRI and fMRI, respectively) studies have been conducted in individuals with FXS (for review, see), differences in frontal lobe structure and function are present (even after controlling for differences in total brain volume) and related to impaired EF in this patient population. For example, individuals with FXS have shown reduced frontal lobe gray [10, 11] and white matter volume [10] as well as reduced activation of frontal regions during EF tasks of working memory and inhibitory control [12, 13].

MRI and fMRI studies in this population are inherently challenging since cardinal features of the disorder (e.g., anxiety, hyperactivity) prevent many full mutation males with FXS from participating, thus limiting the ability to generalize findings. Electroencephalography (EEG) offers a minimally invasive neuroimaging technique that can be more broadly applied in this population while still providing novel insights into the neurophysiological features of frontal lobe dysfunction [14–16]. It also can be more readily synchronously leveraged in preclinical studies using the mouse model of the disorder (for example, see [17]). Our previous EEG studies have demonstrated that individuals with FXS at rest have reduced alpha power, but increased gamma power, which together suggest cortical hyperexcitability [16, 18], a finding that has been replicated in in vivo slice physiology and mouse studies [17, 19–21]. Notably, sex differences in resting state alpha and gamma power differences have emerged in FXS, demonstrating that electrophysiological alterations are more pronounced in males with FXS [15, 22].

In addition, our group [16] has previously studied whole-brain connectivity in different frequency bands using EEG. Individuals with FXS demonstrated reduced inhibitory (alpha band) and enhanced excitatory (gamma band) connections across frontal, parietal, and occipital regions relative to age-matched controls. Similar findings of reduced alpha band connectivity in FXS, including within frontal regions, have been reported by other groups [14, 23]. Yet, likely in part due to its relatively small sample sizes, sex-specific connectivity patterns have not been shown in FXS. Since frontal oscillatory activity has established importance for optimal EF task performance [24–26], increased neural excitability in the frontal cortex may contribute to the cognitive alterations seen clinically in FXS. Of note, our group recently demonstrated a relationship between reduced phase locking to a chirp auditory stimulus and increased flexibility and distractibility errors [27], suggesting an important link between atypical neural dynamics and EF. Still, EEG studies in FXS largely have reported on small samples (*n* < 20) and have not been source-localized, and connectivity features have not been examined in relation to EF performance nor contrasted in male and female patients. Thus, examining phase connectivity within the frontal cortex and its relation to performance-based EF measures may provide novel insight into the functional relevance of altered alpha and gamma band oscillatory activity in FXS.

The present study compared intrinsic resting frontal phase connectivity in individuals with FXS and matched typically developing controls (TDC) and examined the relationship between frontal connectivity and performance-based measures of EF in FXS. We predicted that individuals with FXS would display increased connectivity of gamma oscillations but reduced connectivity of alpha oscillations in frontal cortex compared to TDC, and these findings would be particularly pronounced among males with FXS consistent with our resting power findings. We also predicted that frontal connectivity alterations would be associated with greater EF dysfunction in FXS, even after controlling for general cognitive functioning.

## Methods

### Participants

Sixty-one participants with a genetic diagnosis of FXS (mean age = 21.0, SD = 10.2; age range: 5.9–45.7; 28 females) and 71 age- and sex-matched controls (mean age = 22.2, SD = 10.7; age range: 5.9–48.2; 30 females) participated in study procedures (Table 1). Diagnosis of FXS was confirmed via Southern Blot and polymerase chain reaction (PCR) assays performed at Rush University in the laboratory of Dr. Elizabeth Berry-Kravis. Ten males with mosaicism (methylation or size) were included in the study and in all analyses unless otherwise indicated. FXS participants were excluded from the study if they were being treated for seizures within the past year or taking benzodiazepines, which are known to impact electrophysiological recordings. Several FXS participants were being treated with psychiatric medications at the time of testing, but were on stable dosing for at least six weeks. Twenty-one participants with FXS were receiving stimulants, 14 antipsychotics, and 28 antidepressants. Two controls were receiving stable dosing of SSRIs with no active psychiatric symptoms; removal of these participants from analysis did not result in any substantive changes in results and thus were included in final analyses.

**Table 1:** Participant demographic information

	FXS (*n* = 61)	TDC (*n* = 71)
Age in years	21.0 (10.2)	22.2 (10.7)
Sex (*n*, % male)	33 (54)	41 (58)
IQ deviation score	50.6 (30.9)***	103.2 (9.2)
Nonverbal *Z*-score	− 3.96 (2.5)***	0.23 (0.7)
Verbal *Z*-score	− 2.62 (1.9)***	0.21 (0.9)

Mean (standard deviation) unless otherwise noted

***p < .001

Participants completed the abbreviated Stanford Binet-5th edition (SB-5, [28]) to estimate general cognitive functioning. Standard scores were converted to deviation IQ scores, and scaled scores were converted to z-scores in order to reduce floor effects present for individuals with severe cognitive impairments and to better evaluate inter-individual variability [29]. All participants or their legal guardian, when appropriate, provided written informed consent and assent before participating. The study was approved by Cincinnati Children’s Hospital Medical Center institutional review board.

### Data acquisition

In order to facilitate cooperation during EEG data collection, participants were seated comfortably while watching a silent video as done in previous studies [15, 16, 18, 30]. Five minutes of continuous resting EEG was collected by 128-channel EGI HydroCel Geodesic Sensor Net with a sampling rate at 1000 Hz. As previously described, data were preprocessed by filtering, visual inspection on 2 s epochs and channels, and ICA artifact removal (EEGLAB)[15, 18]. After preprocessing, an average of 125 epochs (SD: 20.8, range 44–187) for FXS and 131 (SD: 18.1, range 43–161) epochs for TDC of artifact-free data remained with no group differences (*p* = 0.10).

### Neural connectivity

As a method recently validated in FXS [22], source-localized time series for each subject were constructed by the minimum norm estimate method in Brainstorm [31]. The reconstructed cortical model consisted of 15,002 vertices which were parcellated into 68 nodes via the Desikan–Killiany atlas [32]. Subsequent analysis steps were focused on eighteen nodes which were designated in frontal regions known contributions to EF [33–35] (Additional file 1).

Time series decomposition was performed via a series of Morlet wavelets where the frequencies included 10.5–12.5 Hz with a step size of 0.5 Hz representing the upper alpha band range and 30–55 Hz with a step size of 5 Hz representing the gamma band range. These bands were selected based on their relevance in previous FXS and EF studies [36–42]. Debiased weighted phase lag index (dWPLI), a measure that quantifies phase lead and lag relationship from a pair of signals [43], was chosen to study frequency-dependent spatial phase synchronization.

dWPLI was selected over other phase connectivity measures for its robustness against volume conduction in hypothesis-driven studies [44, 45]. The dWPLI estimates are negatively biased and has a theoretical range of [− 1, 1], such that zero and negative dWPLI values indicate lack of synchrony and one denotes consistent synchrony between two signals. Properties of the dWPLI measure, including the equation used for dWPLI, are discussed in supplementary materials (Additional file 2). With stationarity assumption from resting state EEG, dWPLI values were averaged over epochs per connection within each participant to characterize overall signal coherence, with an outlier criterion such that values greater than three standard deviations outside their individual mean were rejected.

### Computerized testing of EF

Performance-based EF was estimated using the Test of Attentional Performance for Children (KiTAP) [46], a computerized measure of EF that is reproducible and clinically valid in individuals with FXS [47]. Participants completed four subtests: Alertness (processing speed), Distractibility (attention), Go/NoGo (response inhibition), and Flexibility (cognitive flexibility). Prior to each subtest, participants received verbal instructions and completed a practice task to ensure comprehension. Due to poor comprehension, five individuals with FXS did not complete Distractibility, five did not complete Go/NoGo, and eleven did not complete Flexibility tests. All controls completed subtests with the exception of one who did not complete Go/NoGo due to a technical issue.

A total of eight KiTAP variables were selected for analysis based on a priori hypotheses. Six KiTAP variables have established clinical validity and reproducibility in FXS [47]: Median response times for Alertness (1) and Flexibility (2); Standard deviation of response times for Alertness (3); and Number of errors for Distractibility (4), Go/NoGo (5), and Flexibility (6). An additional two variables, median response times for Distractibility (7) and Go/NoGo (8), were included based on previous studies documenting impaired speed and accuracy trade-off during tasks of cognitive control in neurodevelopmental disorders [3, 48].

### Statistics

Linear mixed effect models (from R library lme4; [49]) were constructed to test for group differences for each connection region and frequency band. Connection regions were pre-defined within and across hemispheres. We grouped connectivity measures as: (1) left prefrontal, (2) right prefrontal, (3) left (posterior) frontal, and (4) right (posterior) frontal. Last, (5) Connections with identical cross-hemispheric nodes also were evaluated. Single region comparisons analyzed connections within that particular region. In each model, the response variable was dWPLI values transformed (Box-Cox 1-parameter) for residual normality. Each model started with all 4 fixed effects (group, sex, connection, and frequency) and a random effect of subject as an intercept. For fixed effect frequency, we used step sizes consistent with time series decomposition for both alpha and gamma frequency bands (alpha: 10.5–12.5 Hz, 0.5 Hz step; gamma: 30–55 Hz, 5 Hz step). The final model was based on a parsimonious principle on the fixed side. To account for differences in general cognitive functioning between groups which may impact connectivity findings, all models also were tested with nonverbal IQ included as a covariate. In FXS, nonverbal IQ is a better representation of overall cognitive functioning and controls for the potential confounding influence of language abilities on this overall score.

Additional models were examined within the FXS group only. First, to further examine whether males with FXS and females with FXS differ from each other as previously implicated by our work [15, 22], group was dropped from our model, but otherwise the model remained the same, including examining with and without nonverbal IQ as a covariate. Second, to determine whether participants using stimulants at the time of testing impacted findings, we added stimulant usage as an additional fixed effect within the model. For all models, maximum likelihood was used for parameter estimation and the Satterthwaite approximation was used for the degrees of freedom. P values were adjusted for multiple pair comparisons using the false discovery rate (FDR).

#### Clinical correlations

To examine the relationship between frontal connectivity and EF, correlations were conducted between connectivity measures and KiTAP variables in participants with FXS. Given the strong association between IQ and EF, and to evaluate EF functions independent from general cognitive ability, partial Spearman correlations were adjusted for nonverbal IQ z-scores in order to limit the effect of impaired intellectual functioning on potential relationships between our neurophysiological and EF measures. This would allow us to interpret any significant corrections as a valid estimate of the relationship of phase connectivity with specific EF domain, independent from general cognitive functioning. Age also was included in partial correlations to account for wide age range sampled. Correlations were calculated across all FXS participants as well as for males and females separately. Due to the ceiling effect on KiTAP in TDC, correlations of EEG features and cognition were not examined in TDC.

#### Regression models

To further characterize the relationship between frontal connectivity and EF in individuals with FXS, generalized linear models were conducted to determine the best dWPLI predictors, if any, for each of the KiTAP scores adjusting for sex, nonverbal IQ, and age. The backward elimination variable selection technique was used to achieve the most parsimonious model. The criterion used to select the variables was based on the corrected Akaike information criterion (AICC), with a variable retained in the model if its *p* value < 0.05. If none of the dWPLI variables were selected, then separate models were examined for males and females with IQ forced in the model. Second-order interaction terms were also examined but none survived the variable selection procedure. Lastly, for each model selected, residuals were examined to see whether a different distribution (and its associated link function) was necessary to achieve a better fit. All correlations and regression modeling were conducted using SAS^®^ software version 9.4 (SAS Institute Inc., Cary, NC).

## Results

### Neural connectivity

#### Gamma band

**Fig. 1:**
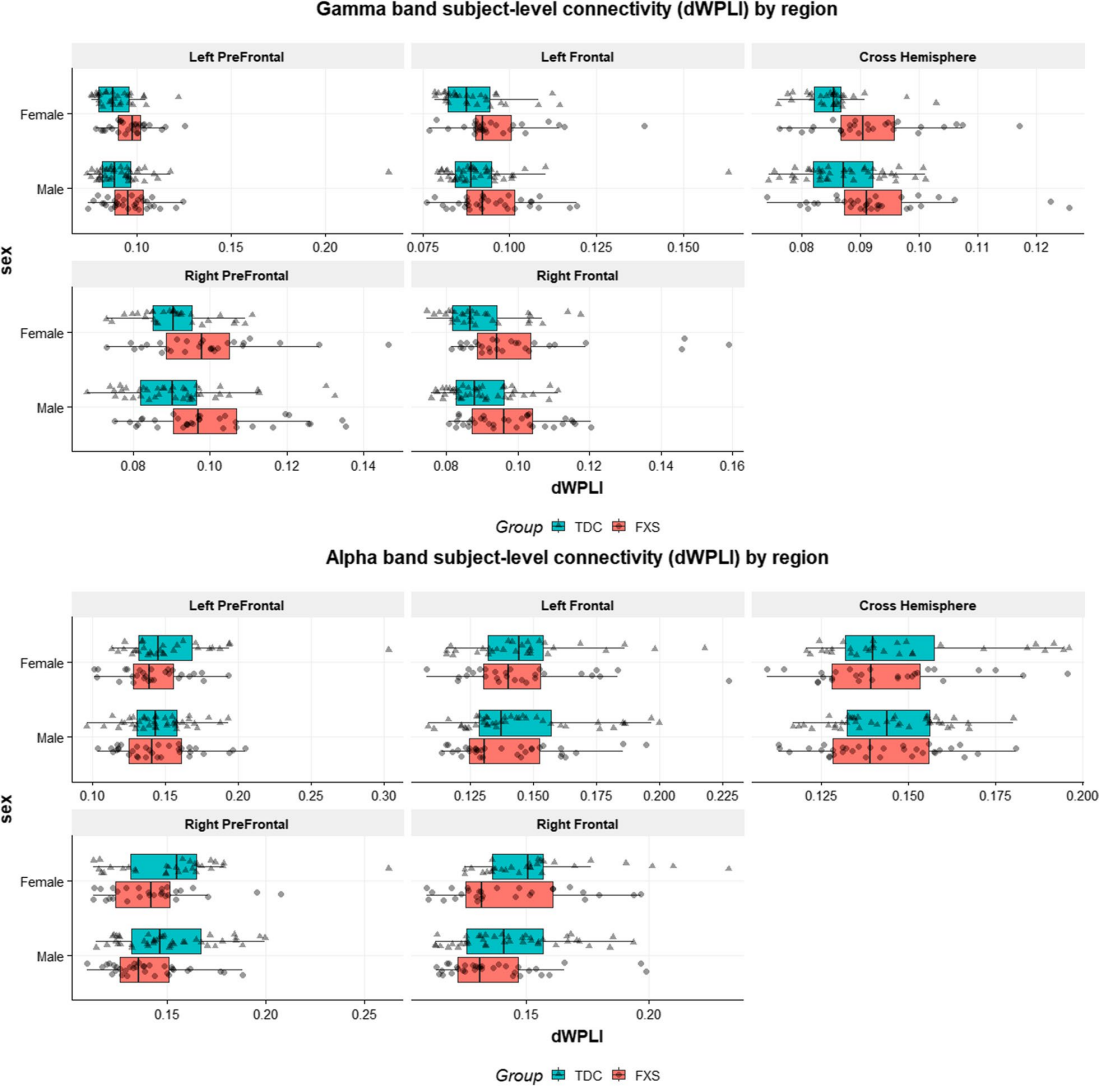
Visualization of frontal connectivity measured by dWPLI across FXS and TDC participants. Each data point represents the average of dWPLI values specific to a region and a frequency band within an individual subject. Boxplots show the 25, 50, and 75 percentiles (lower hinge, median, and upper hinge) per subgroup, separated by group and sex. Any data points beyond the whiskers, which are 1.5 times of IQR from the hinge, are regarded as outliers. Triangle markers denote a typically developing control (TDC), and circle markers denote a participant with Fragile X syndrome (FXS)

**Fig. 2:**
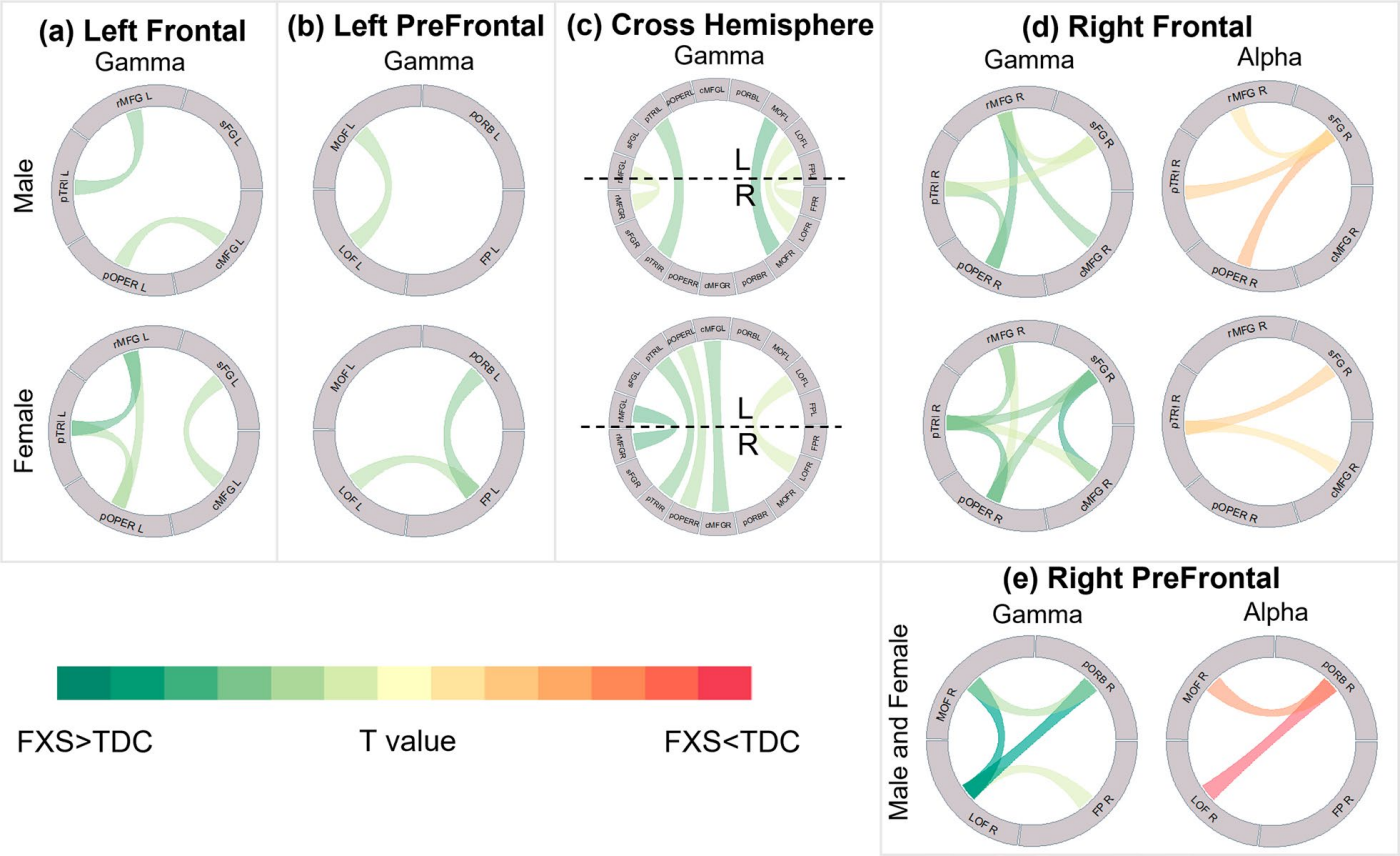
Circular chart of connections highlighting significant group differences in connectivity. Statistically significant group differences (multiple comparisons were FDR corrected per model) for band-specific connectivity depicted in regions **a** left frontal, **b** left prefrontal, **c** cross-hemisphere, **d** right frontal, and **e** right prefrontal. Male and females are shown separately when an interaction with sex was significant. Green ribbon denotes significant FXS > TDC connections, and red ribbon denotes significant FXS < TDC connections. Darker colors represent higher *T*-values. Atlas abbreviations: *cMFG* caudal middle frontal, *FP* frontal pole, *LOF* lateral orbitofrontal, *MOF* medial orbitofrontal, *pOPER* pars opercularis, *pORB* pars orbitalis, *pTRI* pars triangularis, *rMFG* rostral middle frontal, *sFG* superior frontal

A descriptive summary of gamma band functional connectivity (dWPLI) findings is presented in Fig. 1, with connections showing statistical significance for each region represented by color ribbons in Fig. 2. The full list of node pairs with significant case–control differences also is presented in Additional file 3: Tables S1–S5.

**Fig. 3:**
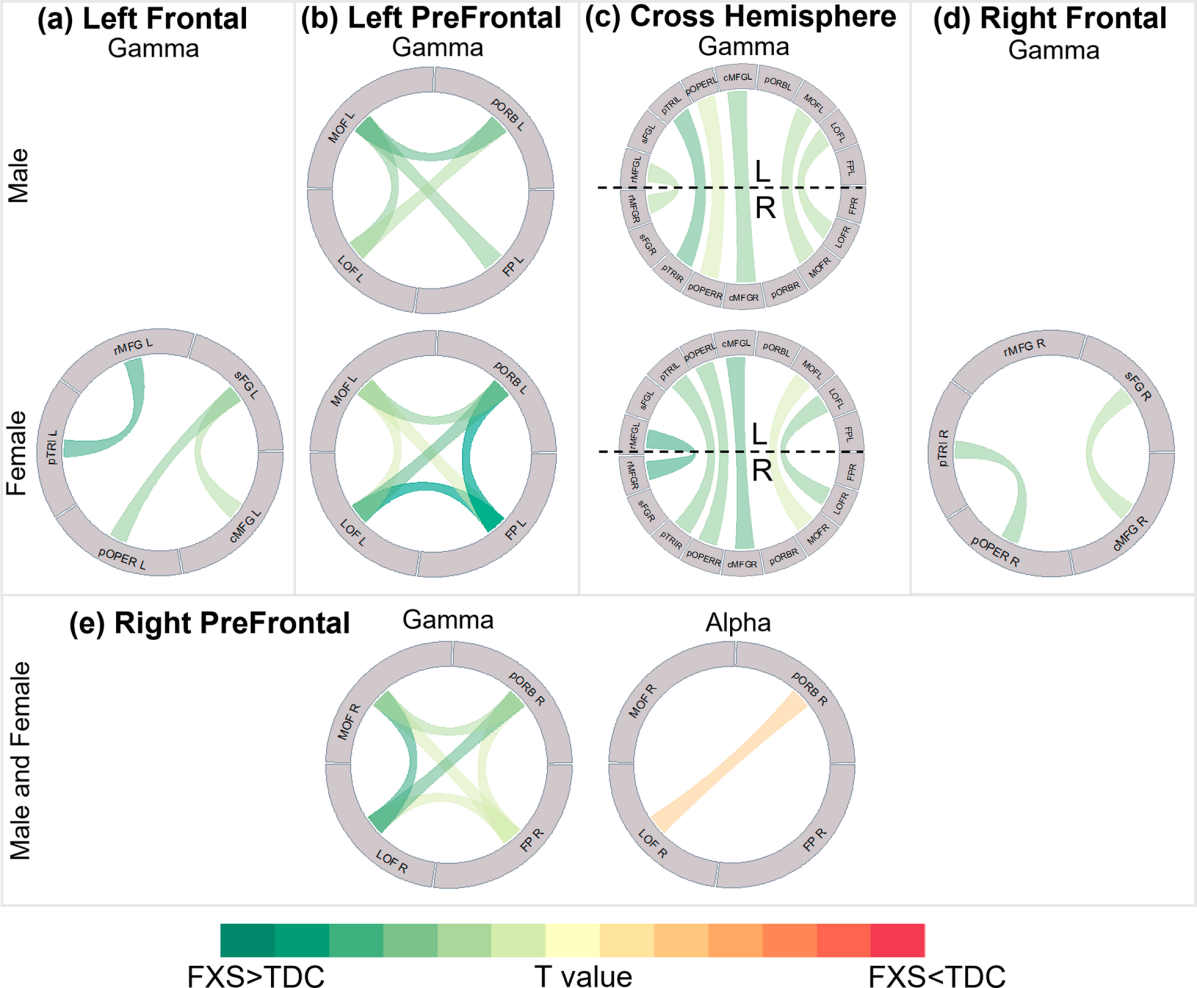
Circular chart of connections highlighting significant group differences in connectivity after accounting for nonverbal IQ. Statistically significant group differences (multiple comparisons were FDR corrected per model) for band-specific connectivity depicted in regions **a** left frontal, **b** left prefrontal, **c** cross-hemisphere, **d** right frontal, and **e** right prefrontal. Male and females are shown separately when an interaction with sex was significant. Green ribbon denotes significant FXS > TDC connections, and red ribbon denotes significant FXS < TDC connections. Darker colors represent higher T values. Atlas abbreviations: *cMFG* caudal middle frontal, *FP* frontal pole, *LOF* lateral orbitofrontal, *MOF* medial orbitofrontal, *pOPER* pars opercularis, *pORB* pars orbitalis, *pTRI* pars triangularis, *rMFG* rostral middle frontal, *sFG* superior frontal

Gamma band functional connectivity was significantly increased in all connections in FXS compared to TDC, indicating widespread elevations across all frontal regions in the patients. Linear modeling of gamma band dWPLI detected significant 3-way interactions (group/sex/connection) for left posterior frontal region (Fig. 3A; *F*(9, 7584.2) = 3.35, *p* = 0.0004), left prefrontal region (Fig. 3B; *F*(5, 4571.1) = 6.63, *p* < 0.0001), right posterior frontal region (Fig. 3D; *F*(9, 7563.0) = 2.98, *p* = 0.002), and cross-hemisphere connections (Fig. 3C; *F*(8, 6948.6) = 2.24, *p* = 0.0219). Post hoc pairwise comparisons of group in the left frontal region indicated that connectivity strength was greater in males with FXS compared to their sex-matched controls in pars opercularis (adj. *p* = 0.019) and pars triangularis (adj. *p* = 0.019). Similar connectivity strength elevations in females with FXS compared to TDC were observed between caudal middle frontal–superior frontal (adj. *p* = 0.019), pars opercularis–pars triangularis (adj. *p* = 0.028), pars opercularis–rostral middle frontal gyrus (adj. *p* = 0.028), and pars triangularis–rostral middle frontal gyrus (adj. *p* = 0.012).

In right prefrontal cortex (Fig. 2E), we detected a 3-way interaction of gamma band activity in group/sex/frequency (*F*(5, 4559.0) = 3.87, *p* = 0.002) and a 2-way interaction of group/connection (*F*(5, 4559.4) = 4.95, *p* < 0.0002). Post hoc pairwise comparisons of group for group/connection showed increased connectivity strength in FXS compared to TDC in frontal pole–lateral orbitofrontal (adj. *p* = 0.0185), lateral orbitofrontal–medial orbitofrontal (adj. *p* < 0.0003), lateral orbitofrontal–pars orbitalis (adj. *p* < 0.0003), and medial orbitofrontal–pars orbitalis (adj. *p* = 0.006) as shown in Additional file 3: Table S4.2. Interestingly, the 3-way interaction of group/sex/frequency showed increased gamma connectivity strengths in males with FXS compared to sex-matched controls in the low gamma band range (30 Hz *p* = 0.04, 35 Hz *p* = 0.0001, 40 Hz *p* = 0.005). Increased connectivity strength also was found in females with FXS compared to their control counterparts in higher gamma band ranges (45 Hz *p* = 0.02, 50 Hz *p* = 0.02, 55 Hz *p* = 0.01).

##### Accounting for IQ

After accounting for nonverbal IQ (Additional file 3: Tables S1–S5), males with FXS no longer showed significantly increased connectivity strength compared to male controls in both left (Fig. 3A) and right (Fig. 3D) frontal regions. However, females with FXS demonstrated increased connectivity strength relative to their TDC counterparts in left frontal regions, specifically in connections caudal middle frontal–superior frontal (adj. *p* = 0.032), pars opercularis–superior frontal (adj. *p* = 0.024), and pars triangularis–rostral middle frontal (adj. *p* = 0.002). In the left prefrontal region (Fig. 3B), significantly increased connectivity strength in FXS relative to TDC remained. Female with FXS had two unique connections frontal pole–lateral orbitofrontal (adj. *p* < 0.0001) and frontal pole–pars orbitalis. In the right frontal region, females with FXS showed increased connectivity strength compared to control females in connections caudal middle frontal–superior frontal (adj. *p* = 0.031) and pars opercularis–pars triangularis (adj. *p* = 0.031). In the right prefrontal region (Fig. 3E), the FXS group demonstrated increased connectivity strength compared to TDC across all connections. In the model for cross-hemisphere connections (Fig. 3C), increased connectivity strength was observed in FXS relative to TDC, though sexes differed slightly in which connections were found to be significant.

##### Accounting for stimulant use

In FXS participants, the use of stimulants did not show any effect on gamma band connectivity strength across connections.

##### Sex differences in FXS

We further investigated group difference between sexes (i.e., male (FXS-TDC)–female (FXS-TDC)) for each EEG frequency, but failed to show significance, indicating that the degree of change in males did not differ significantly from those seen in females. Additionally, when examining the FXS group only, no sex differences emerged for any connection in the post hoc pairwise comparison, even after accounting for nonverbal IQ. To confirm findings, we removed the 10 males with mosaicism, as this subgroup tends to have intermediate phenotypes between males with full mutation FXS and females with FXS. Again, no sex differences were found to be significant in the post hoc pairwise comparison, even after accounting for nonverbal IQ.

#### Alpha band

A subject-level descriptive summary of upper alpha band dWPLI values by group and region is presented in Fig. 1. Connections showing statistical significance in alpha band for each region are represented by color ribbons in Fig. 2. Post hoc group comparisons showed reduced connectivity strength for lateral orbitofrontal–pars orbitalis and medial orbitofrontal–pars orbitalis in FXS relative to TDC across sexes (Additional file 3: Tables S6–7). Upper alpha band functional connectivity was reduced in FXS in right posterior frontal and prefrontal regions relative to controls. Of note, group differences in the upper alpha band were lateralized in the right hemisphere only. In right posterior frontal region (Fig. 2D), we detected a significant group/sex/connection 3-way interaction (*F*(9, 6403.2) = 4.72, *p* < 0.0001). In the post hoc group comparisons, connectivity strength for the pars triangularis–superior frontal connection was reduced in FXS relative to TDC in both sexes. Alpha band right prefrontal region model also detected a significant 2-way interaction of group/connection (Fig. 2E; *F*(5, 3806.2) = 13.43, *p* < 0.0001).

##### Accounting for IQ

After adjusting for nonverbal IQ (Additional file 3: Table S8), group differences in the right frontal region no longer reached significance. However, participants with FXS still demonstrated reduced connectivity strength compared to TDC in the right prefrontal region (Fig. 3E; lateral orbitofrontal–pars orbitalis adj. *p* = 0.0384).

##### Accounting for stimulant use

For participants with FXS, effects of stimulants on alpha band connectivity differed by sex (Additional file 3: Tables S9–11). In general, females taking stimulants demonstrated reduced alpha band connectivity compared to females not taking stimulants. This pattern was observed in the right frontal and the right prefrontal regions (pars opercularis–pars triangularis, adj. *p* = 0.044; lateral orbitofrontal–pars orbitalis, adj. *p* = 0.0232; medial orbitofrontal–pars orbitalis, adj. *p* = 0.0036). In contrast, males taking stimulant showed increased alpha band connectivity compared to males not taking stimulant. Specifically, this finding was observed in the right frontal region (rostral middle frontal–superior frontal, adj. *p* = 0.044), right prefrontal region (lateral orbitofrontal–medial orbitofrontal, adj. *p* = 0.0232) as well as in the cross-hemispheric connections (pars triangularis, adj. *p* = 0.0018).

##### Sex differences in FXS

Within alpha band, males with FXS showed reduced connectivity strength compared to females with FXS subjects at rostral middle frontal among cross-hemispheric connections (adj. *p* = 0.0027). This relationship remained significant after accounting for IQ (adj. *p* = 0.0054). After removing the mosaic males from analysis, the sex difference in the cross-hemispheric connection remained (adj. *p* = 0.0477), but not after accounting for nonverbal IQ (Additional file 3: Table S12).

### Executive function

As expected, results from the KiTAP indicate that individuals with FXS have a lower performance overall across EF tasks as indicated by longer median response time and increased number of errors (Table 2). Across KiTAP variables, participants with FXS taking stimulants at the time of testing did not differ from their FXS counterparts not taking stimulants (Additional file 4: Tables S13–15).

**Table 2:** Summary of KiTAP performance for FXS and TDC participants

	FXS	TDC
**Alertness**		
RT median	618.54 (372)***	344.85 (103)
RT SD	313.03 (344)***	61.70 (44)
**Distractibility**		
RT median	560.63 (227)**	464.10 (766)
Errors	16.23 (13)***	5.34 (7)
**Flexibility**		
RT median	1168.38 (652)***	699.79 (262)
Errors	9.34 (5)***	0.83 (1)
**Go/NoGo**		
RT median	492.55 (140)*	444.39 (82)
Errors	4.61 (6)***	0.8 (2)

All values given in mean (standard deviation)

*RT* reaction time, *SD* standard deviation

*p* value: *< 0.05, **< 0.01, ***< 0.001

#### Relationships between neural connectivity and executive function

##### Spearman correlations

No significant correlations between IQ and dWPLI values in either frequency band were found in FXS.

**Fig. 4:**
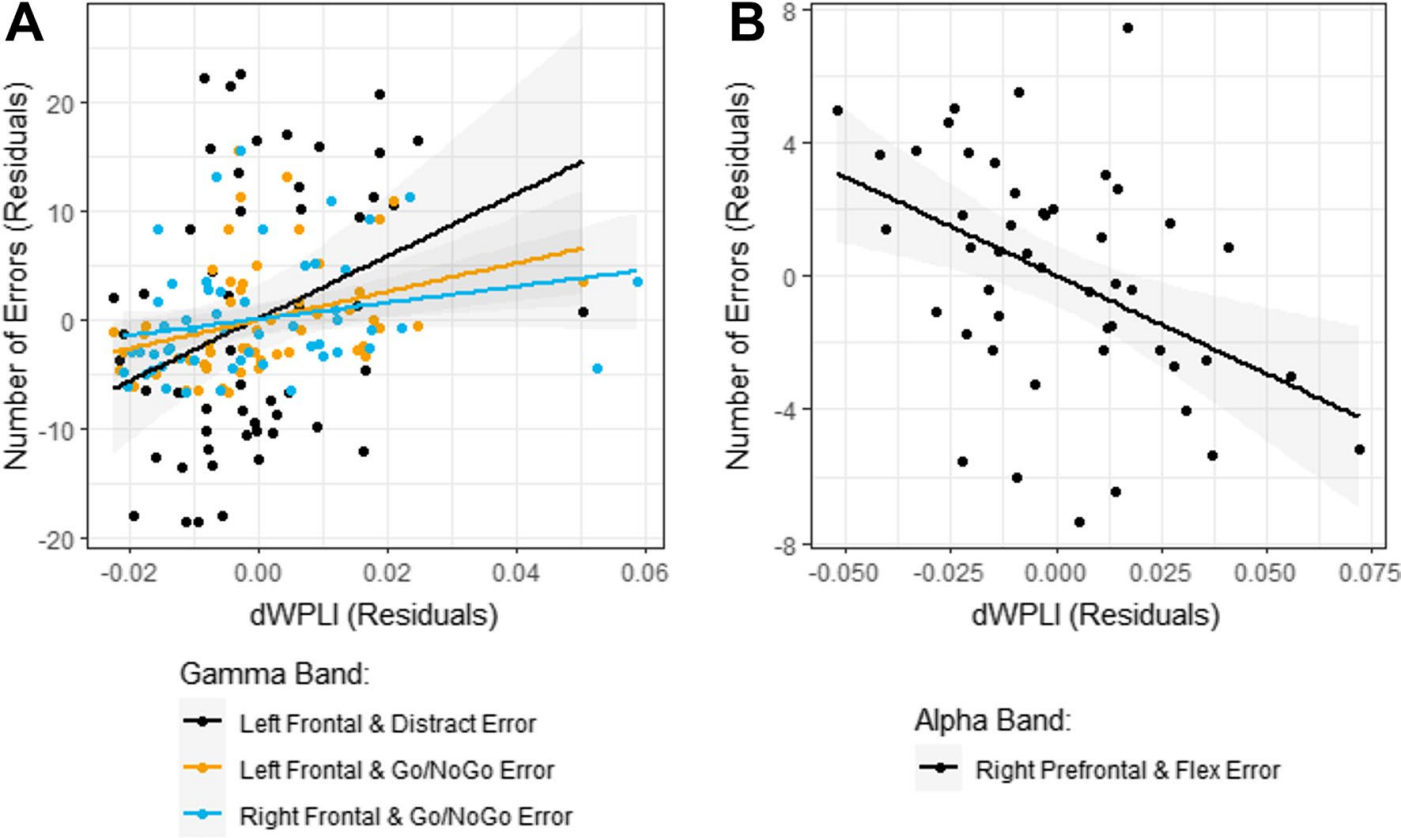
Correlations between frontal connectivity and executive function measures across FXS. Spearman’s correlations showing positive correlation in the gamma band (**A**) and negative correlation in alpha band (**B**) between connectivity strength and number of errors

Table 3 gives significant Spearman correlations between dWPLI and KiTAP variables adjusted for nonverbal IQ and age (see Additional file 5: Tables S16–17 for all correlations). Across FXS participants, we found significant relationships between increased error rates on the Distractibility and Go/NoGo tasks and increased gamma band connectivity strength in left posterior frontal and right posterior frontal regions (Fig. 4A). In alpha band, we found that reduced error rate during Flexibility was related to increased dWPLI connectivity in right prefrontal regions (Fig. 4B).

**Table 3:** Significant Spearman’s partial correlations (accounting for nonverbal IQ and age) between connectivity strength and executive function in FXS

Frequency band	Regional comparison	KiTAP variable	FXS group	rho	*p*
Gamma	Left frontal	Distractor error	All	0.35	0.009
			Male	0.57	0.003
	Left frontal	Go/NoGo error	All	0.45	0.001
			Male	0.58	0.002
	Left frontal	Flexibility median RT	Male	− 0.54	0.01
	Right frontal	Go/NoGo error	All	0.32	0.02
			Male	0.49	0.01
	Right prefrontal	Flexibility error	Female	− 0.52	0.01
	Cross-hemisphere	Flexibility error	Female	− 0.46	0.03
Alpha	Right prefrontal	Flexibility error	All	− 0.32	0.03

**Fig. 5:**
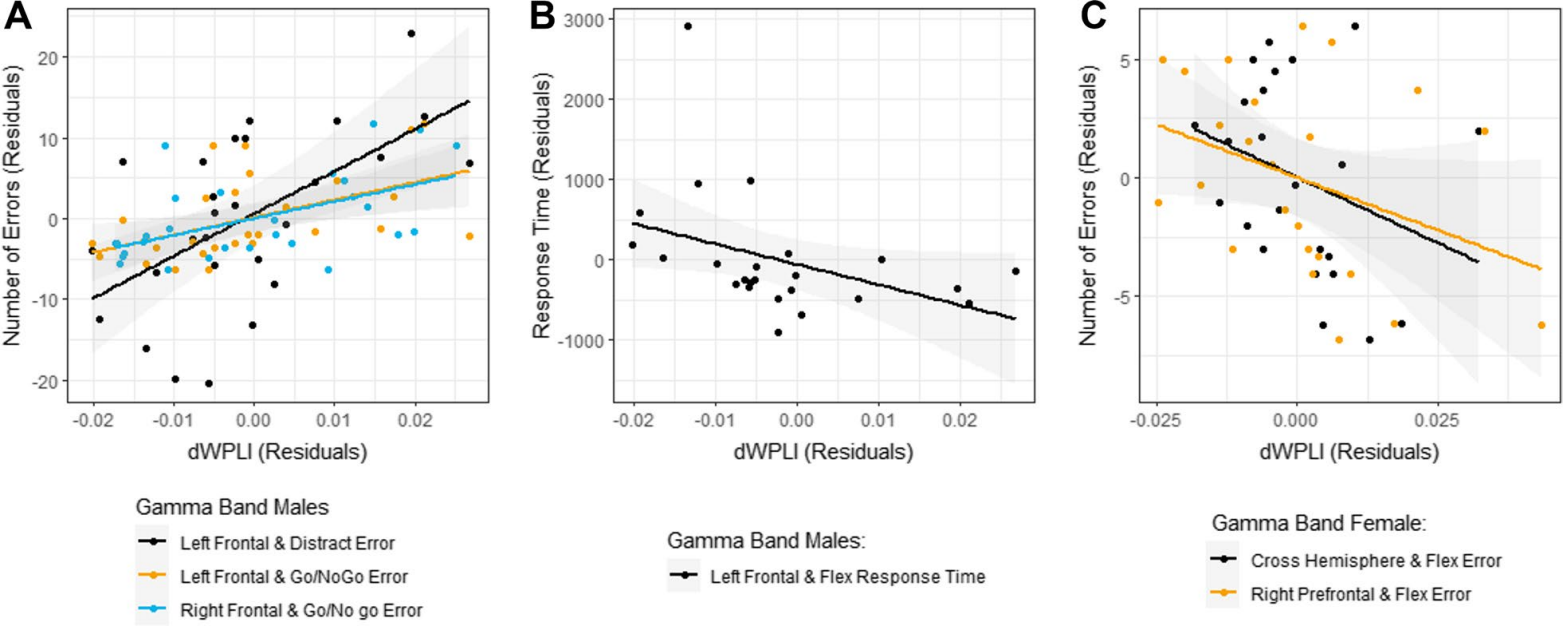
Correlations between frontal connectivity and executive function measures by sex. Sex-specific significant Spearman’s correlations are shown separately for males (**A**, **B**) and females (**C**) for both gamma and alpha bands

Relationships between gamma band connectivity and error rates were primarily driven by males with FXS (Fig. 5A). We also found that increased connectivity strength in the gamma band was associated with shorter response times during the Flexibility task in males with FXS (Fig. 5B). Unexpectedly, in females with FXS, we found that increased gamma band cross-hemispheric and right prefrontal connectivity strength were associated with fewer Flexibility errors (Fig. 5C).

##### Regression models

Significant regression models are provided in Table 4, which give the best fitting models for KiTAP scores as a linear function of selected dWPLI measures after controlling for sex, age, and nonverbal IQ. For each model, the band-specific dWPLI measure served as the dependent factor. For each significant model included in Table 4, if no distribution is noted, then the default Gaussian model was used. If a distribution is noted, then its residual profile is a better fit.

**Table 4:** Significant regression models in FXS

Frequency band	KiTAP variable	Effect	Estimate	SE	*DF*	*t*	*p*
Gamma (Poisson)	Distractor error	Intercept	0.870	0.254	50	3.42	0.001
		Sex (F)	0.092	0.081	50	1.13	0.263
		Nonverbal *Z*-score	− 0.088	0.018	50	− 4.82	< 0.0001
		Age	− 0.003	0.004	50	− 0.80	0.426
		dWPLI Left frontal	15.562	2.251	50	6.91	< 0.0001
Gamma (Log normal)	Flexibility median RT	Intercept	8.173	0.488	43	16.77	< 0.0001
		Sex (F)	0.134	0.172	43	0.78	0.440
		Nonverbal *Z*-score	− 0.059	0.0338	43	− 1.75	0.086
		Age	− 0.019	0.007	43	− 2.69	0.010
		dWPLI Left frontal	− 11.218	4.544	43	− 2.47	0.018
Gamma (female only)	Flexibility error	Intercept	16.171	6.813	19	2.36	0.029
		Nonverbal *Z*-score	− 0.966	0.392	19	− 2.46	0.024
		Age	− 0.128	0.086	19	− 1.48	0.156
		dWPLI Left frontal	174.93	78.772	19	2.22	0.039
		dWPLI Cross-hemi	− 294.98	108.36	19	− 2.72	0.014
Alpha	Flexibility error	Intercept	14.46	3.252	43	4.44	< 0.0001
		Sex (F)	− 1.025	1.259	43	− 0.81	0.420
		Nonverbal *Z*-score	− 1.204	0.260	43	− 4.64	< 0.0001
		Age	− 0.053	0.053	43	− 1.00	0.321
		dWPLI Right prefrontal	− 60.564	18.245	43	− 3.32	0.002
Alpha (Poisson)	Distractor error	Intercept	3.463	0.222	50	15.61	< 0.0001
		Sex (F)	0.242	0.081	50	2.98	0.005
		Nonverbal *Z*-score	− 0.082	0.019	50	− 4.37	< 0.0001
		Age	0.0002	0.004	50	0.06	0.954
		dWPLI Right frontal	− 8.893	1.610	50	− 5.52	< 0.0001

Briefly, in the gamma band, for every 0.01 increase in dWPLI in the left frontal region, Distractor error increased by approximately 16%, and Flexibility median reaction time decreased by approximately 10%. The latter is the only model in which age was significant, such that each one year increase in age, Flexibility median reaction decreases by approximately 1.9%. For females only, every 0.01 increase in dWPLI in the left posterior frontal region corresponds to an increase in 1.75 errors during Flexibility, and every 0.1 increase in cross-hemispheric (i.e., more rightward) dWPLI corresponds to a decrease in 2.95 errors during Flexibility. Further, each 0.01 increase in dWPLI in the right prefrontal region in alpha band corresponded to a decrease in 0.61 errors during the Flexibility task, and every 0.01 increase in dWPLI in the right posterior frontal region Distractor error decreased by approximately 9%.

## Discussion

Resting state EEG phase connectivity within source-localized frontal regions in a well-powered sample of individuals with FXS and matched controls revealed two key findings. First, individuals with FXS broadly demonstrated increased gamma and reduced alpha phase connectivity across frontal regions, both within and across hemispheres, compared to TDC. Second, significant associations between EF and frontal connectivity emerged in FXS, such that increased error rates were associated with increased gamma connectivity strength and inversely associated with reduced alpha connectivity strength. Notably, these correlational findings remained robust after accounting for general cognitive functioning and age. We document an important and not previously reported link between deficits in EF and the alterations in the coherence of specific frequencies of neural oscillations within the frontal cortices of individuals with FXS. Together, our study reveals a potential underlying neurophysiological basis for EF impairment in FXS that may represent a promising target for future intervention studies.

### Phase connectivity

Phase connectivity, when applied spatially, assesses the precise alignment of neural oscillations at a specific frequency between brain regions. Phase connectivity, or coherence, between different brain regions is well known to support cognitive functions [43, 45, 50]. Herein, we implemented source localization to examine point-to-point connectivity within frontal regions to enhance regional specificity of previously reported phase connectivity alterations and their functional significance [14, 16, 23]. Previous investigations into other neurodevelopmental and neurological disorders have linked alterations in resting frontal phase connectivity with impaired cognitive function, including deficits in EF [51–53].

Our primary results are consistent with our and other’s previous electrode-level phase connectivity findings, but further localize a subset of regions with gamma band hyper-connectivity and alpha band hypo-connectivity within frontal cortex in individuals with FXS [14, 16, 23]. Specifically, gamma hyper-connectivity and alpha hypo-connectivity may reflect poor top-down regulation of local frontal circuits leading to hyperexcitability of local circuit function and subsequent cognitive and behavioral dysfunction [54, 55]. The observed alpha hypo-connectivity may represent deficient longer-range inhibitory mechanisms which down-regulate background neural excitability [42]. From the perspective of an excitatory–inhibitory imbalance (E:I) model of neurodevelopmental disorders, our current findings are consistent with a hyper-excitable phenotype that has been repeatedly documented in FXS across in vivo slice physiology and mouse model studies [17, 19, 21, 56].

More recently, a growing body of the literature has raised the importance of increased variability in neural signals linked to enhanced cognition, as systems need to be tuned on-line to optimize them for behavioral demands [57–59]. Our observations, when taken together with other EEG findings in FXS (e.g., decreased peak alpha frequency, decreased neural synchronization to the auditory chirp, reduction in global alpha power with concomitant increases in regional gamma power, reduced EEG signal complexity), suggest a diminished capacity or increased constraints on the expression of neural variability in the FXS cortex [15, 22, 60–63]. Elevated gamma band connectivity and suppressed alpha band connectivity at rest, as found here in individuals with FXS, could reflect a neural state patients are seemingly “stuck” in, thus resulting in reduced ability to dynamically adjust neural responses to environmental demands, especially those requiring higher cognitive skills. From a molecular standpoint, loss of FMRP results in a reduction of synaptic plasticity, deficits in stimulus-induced synaptic protein synthesis, synaptic overgrowth, and changes in dendritic spine morphology in the *FMR1*^−/−^ KO mouse and neurons derived from FXS patients [64–68]. Such changes also would be predicted to dampen neural variability at the molecular and cellular levels [60]. Since our findings were relatively similar across sexes in FXS, this suggests a general reduction in FMRP, as opposed to a complete loss, may be sufficient to alter frontal connectivity and, in turn, EF.

### Evolving model of EF physiology in FXS

The association between EF task performance and frontal lobe phase connectivity in the present study can be used to advance our understanding of higher-order cognitive processes in FXS and, for the first time, establish a neurophysiological model of impaired EF in this patient population. Importantly, the correlation and regression findings remained robust, even after correcting for general intellectual functioning and age. This suggests that frontal gamma hyper-connectivity and alpha hypo-connectivity may be specifically related to EF deficits rather than intellectual or general cognitive capacity more broadly. Alterations to alpha and gamma phase connectivity predicted increased error rates during a distractibility task as well as increased error rates and reduced reaction time during a cognitive flexibility task. Although faster reaction times are often thought to indicate more intact cognitive processes, this is not necessarily the case in the context of EF when slower reaction times can be beneficial in terms of the speed/accuracy trade-off [48, 69].

We speculate that functional consequences of connectivity abnormalities may include poorer local regulation of frontal activity in combination with deficient inhibitory mechanisms, thus leading to difficulty in cognitive flexibility, attention shifting, ignoring distractions, and increased impulsivity. Moreover, our findings are consistent with the canonical role of alpha oscillations in attention and cognition, such that enhanced alpha frontal connectivity helps facilitate shifting attention and cognitive resources to support behavioral flexibility and inhibit distraction from sensory information that is not relevant to contextual demands [37, 42]. Thus, our findings suggest increased alpha band connectivity may support EF in a compensatory fashion. Previous fMRI studies have implicated compensatory mechanisms of increased activation in prefrontal regions to support inhibitory control and prevent distractor interference in FXS [13, 70]. Complementing the general activation finding from fMRI work, our EEG study highlights the breadth and spatial distance of altered coherence of neural oscillation across regions that occurs in specific frequency bands that have their own functional significance.

It is important to note that several of our group differences in gamma band connectivity were no longer significant after accounting for nonverbal IQ, which seemed to be particular to males with FXS in right and left frontal regions. As prefrontal regions are more synonymous with their role in EF, this suggests prefrontal gamma hyper-connectivity and alpha hypo-connectivity may be more specific to FXS pathology rather than that associated with general intellectual disability. In contrast, group differences in gamma band connectivity in posterior frontal regions may be more dependent on FMRP expression, which we know are significantly different in FXS and TDC males and correspond to IQ score in FXS males [71]. Future studies examining the relationship between resting state connectivity and FMRP levels are needed to clarify this finding.

### Sex differences *not* observed in FXS

Contrary to our expectations, frontal gamma hyper-connectivity and alpha hypo-connectivity were mostly similarly effected in males and females with FXS. As full mutation males with FXS have significantly less expression of FMRP [71] and a higher burden of clinical symptoms [4], we had predicted connectivity alterations would be more prominent in males than females. Previous studies have replicated the finding of increased resting local gamma power in males with FXS [15, 22, 60, 72]. However, it is important to note that phase-based measures, such as dWPLI, can show increases in phase synchronized neural oscillations across regions rather than just a parallel increase in the power at a given frequency band. Thus, our finding indicate high-frequency activity in local circuits may be restricted to males with FXS, whereas high-frequency activity in the mutual synchronized driving of excitability across widely distributed brain regions may be more broadly present across males and females with FXS.

Interestingly, sex differences in FXS emerged when comparing participants taking stimulants at the time of testing versus those who were not. Males with FXS taking stimulants had *increased* alpha band connectivity in a few connections within frontal and prefrontal regions compared to males taking not stimulants. In contrast, females with FXS taking stimulants had *reduced* alpha band connectivity compared to females not taking stimulants. One possible interpretation of this finding is that stimulants may help “normalize” alpha band connectivity in males with FXS. Given that partial correlations showed increases in alpha band connectivity corresponding to reductions in errors during the Flexibility and Distractibility tasks, especially among males, our findings implicate a possible mechanism by which stimulant medication reduces impulsivity in FXS males.

Although the majority of ADHD studies have not found a difference in frontal connectivity based on whether participants were taking stimulants or not [73–75], at least one study reported frontal connectivity in the alpha band was reduced within the stimulant group compared to the non-stimulant group, consistent with our findings in females with FXS [76]. Thus, it is possible that stimulants may suppress alpha band connectivity within females with FXS. Alternatively, it is possible that females with FXS who take stimulants have lower alpha band connectivity at baseline than females who do not, and the stimulants themselves do not change alpha band connectivity. Future studies are needed to determine the impact of stimulant medication on frontal functional connectivity and EF in FXS.

### Evidence of atypical lateralization

Lateralized substrates for distinct cognitive functions within frontal cortex have been consistently observed in typically developing individuals. For example, right inferior and superior frontal gyri have been implicated in inhibitory control, specifically proactive control related to reaction time slowing [77, 78]. Atypical brain lateralization of cognitive functions has been observed in other neurodevelopmental disorders, including ASD [79, 80]. Our findings add to these previous studies by documenting atypical lateralization in individuals with FXS. For example, we found increased gamma band, but reduced alpha band, in right prefrontal regions in individuals with FXS compared to TDC. This finding suggests EF skills lateralized within these regions would be affected, which our partial correlation and regression findings support. Specifically, we observed reduced alpha connectivity within these atypically lateralized regions predicted impaired performance during cognitive flexibility and distractibility tasks. Yet, consistent with our compensatory hypothesis, our findings also suggest that preserved lateralized right frontal alpha connectivity may facilitate inhibition of previously learned behavior (flexibility) and irrelevant sensory stimuli (distractibility) in individuals with FXS, especially among females. This interpretation is consistent with our recent finding showing preserved lateralization of low gamma activity was related to reduced inhibition errors [27].

Notably, among males with FXS, we found gamma hyper-connectivity in left pars opercularis and pars triangularis, areas within the inferior frontal gyrus which are critical for speech and language. This suggests increased phase synchronized gamma activity within these frontal regions may contribute to language impairments and delays in FXS, which are nearly universal among male patients [81, 82]. Our previous work has shown that prior to the onset of speech production individuals with FXS demonstrate increased frontal gamma power compared to controls, and greater increases in this phasic gamma power were associated with more unintelligible speech in FXS [83]. Yet, this finding in males was no longer significant after controlling for nonverbal IQ. Thus, taken all together, these findings implicate increased local and synchronized high-frequency frontal activity may have widespread disruptive role in FXS that is not necessarily specific to EF or speech production. Future studies are needed to determine the extent to which high-frequency activity within frontal cortices more broadly affects learning and development in FXS.

### Limitations

Frontal connectivity findings are limited to brain activity at rest and thus should not be equated with task-based connectivity findings. Similarly, resting connectivity findings may not generalize to neural activity during real world function. Thus, future work is needed to study dynamic changes in neural oscillation during EF task performance. Findings further are limited to short-range frontal connectivity and do not consider longer-range connectivity relevant to EF (e.g., fronto-parietal connections). Still, findings remain the first of their kind in FXS and represent a critical step to better understanding neurophysiological mechanisms underlying impaired EF in FXS. It also is important to note that only certain aspects of EF were measured using KiTAP, indicating the need to replicate findings in a broader battery of neuropsychological tests (e.g., NIH Cognitive Toolbox). We did not have an IQ- and age-matched control group, and although the majority of our findings remained after controlling for IQ, inclusion of such a control group in addition to examining relationships with FMRP levels is needed in future studies to determine the extent to which our findings are specific to FXS pathology. Similarly, we note that FMRP expression is not dichotomous based on sex as presently described. Thus, future work examining frontal connectivity in relation to a continuous measure of FMRP [71] is needed to better understand the role of FMRP in EF impairments in FXS. Our use of a validated source localization method without the use of MRI was a strength of our study; however, to confirm regional findings future studies would benefit from concomitant MRI acquisition. Another strength of our study was the inclusion of a wide age range of participants; however, we did not have sufficient data across ages to include as an additional factor in our primary connectivity models. Future studies with larger samples are needed to explore the developmental trajectory of frontal functional connectivity and executive function skills in FXS. Last, due to the significant overlap of EF impairments in FXS and ADHD, it also will be important in future work to determine whether frontal functional connectivity patterns differ between individuals with FXS who have comorbid ADHD or not.

## Conclusions

In the first study of its kind using high-density source-localized resting state EEG, we documented increased gamma band connectivity and reduced alpha band connectivity in frontal brain regions in individuals with FXS relative to TDC, and these connectivity abnormalities were predictive of executive function deficits in FXS, independent of general cognitive ability. Our findings implicate gamma hyper-connectivity within frontal brain regions and thus support and extend previous findings demonstrating E:I imbalance in FXS. Given the directions of correlation, we hypothesize that increased gamma connectivity may impair EF performance via its relation to hyperexcitability of cortex, whereas increased alpha connectivity may provide compensatory support for EF in individuals with FXS by facilitating adaptive shifts is brain state needed for context-relevant behavioral demands. Together, our findings provide novel insight into potential mechanisms of deficit in EF in FXS and suggest that frontal phase connectivity may be an important measure of target engagement in future intervention trials.

## Supplementary Information

**Additional file 1.** A priori designated eighteen nodes within frontal regions used for analysis based on their known contributions to executive function.

**Additional file 2.** Properties of dWPLI measure.

**Additional file 3.** Post hoc pair comparisons of group from lme models for gamma and alpha band connectivity.

**Additional file 4.** Summary of KiTAP performance of individuals with FXS taking stimulants (FXS+stimulant), those not taking stimulants (FXS-stimulant), and typically-developing controls (TDC).

**Additional file 5.** Spearman’s Partial Correlations (Accounting for Nonverbal IQ and Age) Gamma Connectivity Strength and Executive Function as well as Alpha Connectivity Strength and Executive Function in FXS.

## Data Availability

The dataset used for the current is available from the corresponding author on reasonable request.

## Abbreviations

ASD: Autism spectrum disorder
CGG: Cysteine-guanine-guanine
CH: Cross-hemisphere
cMFG: Caudal middle frontal
dWPLI: Debiased weighted phase lag index
EEG: Electroencephalography
EF: Executive function
E:I: Excitatory/inhibitory
FP: Frontal pole
FMR1: Fragile X messenger ribonucleoprotein 1 (*FMR1*)
fMRI: Functional magnetic resonance imaging
FMRP: Fragile X protein
FXS: Fragile X syndrome
IAPF: Individual alpha peak frequency
ID: Intellectual disability
IQ: Intelligence quotient
KiTAP: Test of Attentional Performance in Children
LF: Left Frontal
lme: Linear mixed effect
LOF: Lateral orbitofrontal
LPF: Left prefrontal
MOF: Medial orbitofrontal
MRI: Magnetic resonance imaging
PCR: Polymerase chain reaction
pORB: Pars orbitalis
pOPER: Pars opercularis
pTRI: Pars triangularis
RF: Right frontal
rMFG: Rostral middle frontal gyrus
RPF: Right prefrontal
RT: Response time
SB-5: Stanford-Binet-5th Edition
SD: Standard deviation
sFG: Superior frontal
TDC: Typically developing control
