# Supplementary figures and images for: Altered frontal connectivity as a mechanism for executive function deficits in fragile X syndrome
Source: Mol Autism. 2022 Dec 9;13:47. doi: 10.1186/s13229-022-00527-0 (PMC9733336; doi:10.1186/s13229-022-00527-0)

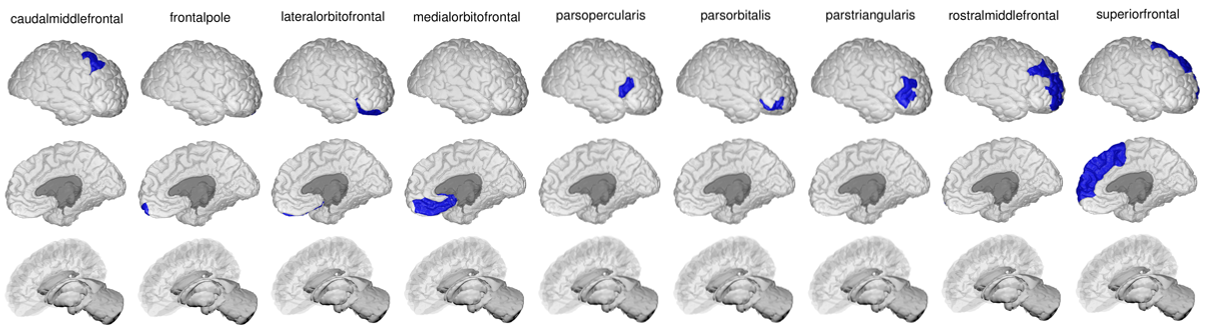

Supplement: Supplementary file 1 — Additional file 1. A priori designated eighteen nodes within frontal regions used for analysis based on their known contributions to executive function. [file 13229_2022_527_MOESM1_ESM.png]
