# Supplementary material for: Altered frontal connectivity as a mechanism for executive function deficits in fragile X syndrome
Source: Mol Autism. 2022 Dec 9;13:47. doi: 10.1186/s13229-022-00527-0 (PMC9733336; doi:10.1186/s13229-022-00527-0)
Supplement: Supplementary file 2 — Additional file 2. Properties of dWPLI measure. [file 13229_2022_527_MOESM2_ESM.docx]

Let x(t) and y(t) be 2 time series from a pair of hypothetically connected nodes for a given epoch t=1:N, their instantaneous analytic representations via wavelet transform are matrices $W^{X}(t,f)$ and $W^{Y}(t,f)$. For simplicity, we discuss the connectivity at a sample frequency $f_{0}$ only, and the corresponding complex-valued vectors are $W^{X}(t,f_{0})$ and$W^{Y}(t,f_{0})$. The complex-valued cross spectrum via wavelet is obtained by$W^{XY}\left( t,f_{0} \right)=W^{X}\left( t,f_{0} \right)\bar{W^{Y}(t,f_{0})}$ (1). Elements in $W^{XY}$can be rewritten as $Re^{i\theta}$ or A+iB where $\theta$ represents the local relative phase difference and B denotes the imaginary part of corresponding XWT. The rotation of the relative phase difference $\theta$ on the unit circle can be reflected on the growth of imaginary part B. For a $\theta$ holds $\pm90$ degrees, its corresponding B reaches its maximal $R$; for another $\theta$ points to 0 or 180 degrees which indicates volume conduction appearance, B equals zero. By transition from pure phase difference $\theta$ to B, the relative phase differences caused by volume conduction are penalized more than by natural brain activities (Figure 1 panel D in Vinck 2011).

Debiased WPLI (dWPLI) corrects the sample size bias by subtracting self-product terms from both numerator and denominator of the squared weighted phase lag index estimator.

$dWPLI=\frac{\left( \sum_{k=1}^{N} B_{k} \right)^{2}-\sum_{k=1}^{N} {B_{k}}^{2}}{\left( \sum_{k=1}^{N} {|B}_{k}| \right)^{2}-\sum_{k=1}^{N} {{|B}_{k}|}^{2}}=\frac{\sum_{i=1}^{N} \sum_{j=i+1}^{N} B_{i}B_{j}}{\sum_{i=1}^{N} \sum_{j=i+1}^{N} \left| B_{i} \right|\left| B_{j} \right|}$

$\mathrm{Given} B_{i},B_{j}\in\mathbf{R}, B_{i}B_{j}=\left\{ \begin{matrix} \left| B_{i} \right|\left| B_{j} \right| \\ -\left| B_{i} \right|\left| B_{j} \right| \end{matrix} \right.\underset{\to}{yields}$ 2 extreme cases: $\left\{ \begin{aligned} \sum_{i=1}^{N} \sum_{j=i+1}^{N} B_{i}B_{j}=\sum_{i=1}^{N} \sum_{j=i+1}^{N} \left| B_{i} \right|\left| B_{j} \right| \\ \sum_{i=1}^{N} \sum_{j=i+1}^{N} B_{i}B_{j}\underset{\to}{binary coloring}-\sum_{i=1}^{N} \sum_{j=i+1}^{N} \left| B_{i} \right|\left| B_{j} \right| \end{aligned} \right.$

$\therefore dWPLI\in[-1,1]$ (dWPLI estimator is negatively biased)

**Discussion**

1) **same-sign** $\boldsymbol{B}_{\boldsymbol{i}}$**s only**: any pair products $B_{i}B_{j}$ are identical to their normalization counterparts $\left| B_{i} \right|\left| B_{j} \right|$ and lead dWPLI approaching its upper bound 1. Ideally, this happens when 2 time series consistently keep instantaneous phase differences concentrated and away from 0 or 180 degree.

However, as long as the instantaneous phase differences distribute on one side of the real axis, dWPLI will always acquire 1 (**Figure S2**) and not reflect the spread or center of the distribution.


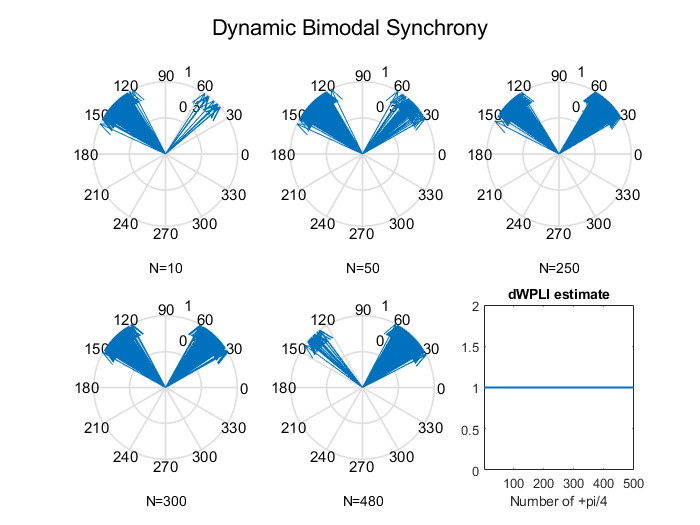


**Figure S2.** A total of 500 simulated phase difference angles are located around 2 centers $\frac{\pi}{4}$ and $\frac{3\pi}{4}$. No matter the sample sizes change between the two groups, dWPLI estimate remains 1. N denotes the sample size of the $\frac{\pi}{4}$ group.

2) **opposite-sign** $\boldsymbol{B}_{\boldsymbol{i}}$**s only**: this case is only for time series of N=2 non-zero or $\pi$ data points, where$B_{1}B_{2}=-\left| B_{1} \right|\left| B_{2} \right|$, dWPLI achieves its lower bound of -1. This atypical example could be from a highly volume-conduction contaminated pair of time series. 4-color theorem prohibits dWPLI=-1 for N>2 non-0 or $\pi$ time series.

3) **same-sign and opposite-sign** $\boldsymbol{B}_{\boldsymbol{i}}$**s:**

In the more general case, where the normalized products $\frac{B_{i}B_{j}}{\sum_{i=1}^{N} \sum_{j=i+1}^{N} \left| B_{i} \right|\left| B_{j} \right|}$ will partially cancel with each other (depending on sign and magnitude of the products), dWPLI ranges between -1 and 1.

Let the sample size holds constant (N=500), two bimodal cases are simulated in **Figure S3**. (a) if the heights of positive and negative $B_{i}$s are comparable, as the sample size of one group increases, dWPLI estimations first drop then increase and form a convex; when the two groups’ sample sizes are close, the lowest estimate appears around zero as an indicator of lack of synchrony, however this estimate is negatively biased. (b) If the heights of positive and negative $B_{i}$s are incomparable (Figure S2 bottom panels), the convex remains and the lowest estimate appears closer to the higher magnitude ${|B}_{i}|$ group.

A common example of lack of phase synchrony is 2 time series’ instantaneous phase difference angles are randomly distributed on the unit circle. **Figure S4** simulation shows dWPLI estimates for this case converge to zero.

The last simulation is about volume conduction. 500 simulated phase difference angles are centered either on 0 or 180 degrees with 0-mean 0.1 in radians-standard deviation Gaussian noise. As the samples shift from unimodal to bimodal and to the other unimodal, dWPLI estimates remain close to zero and negatively biased.


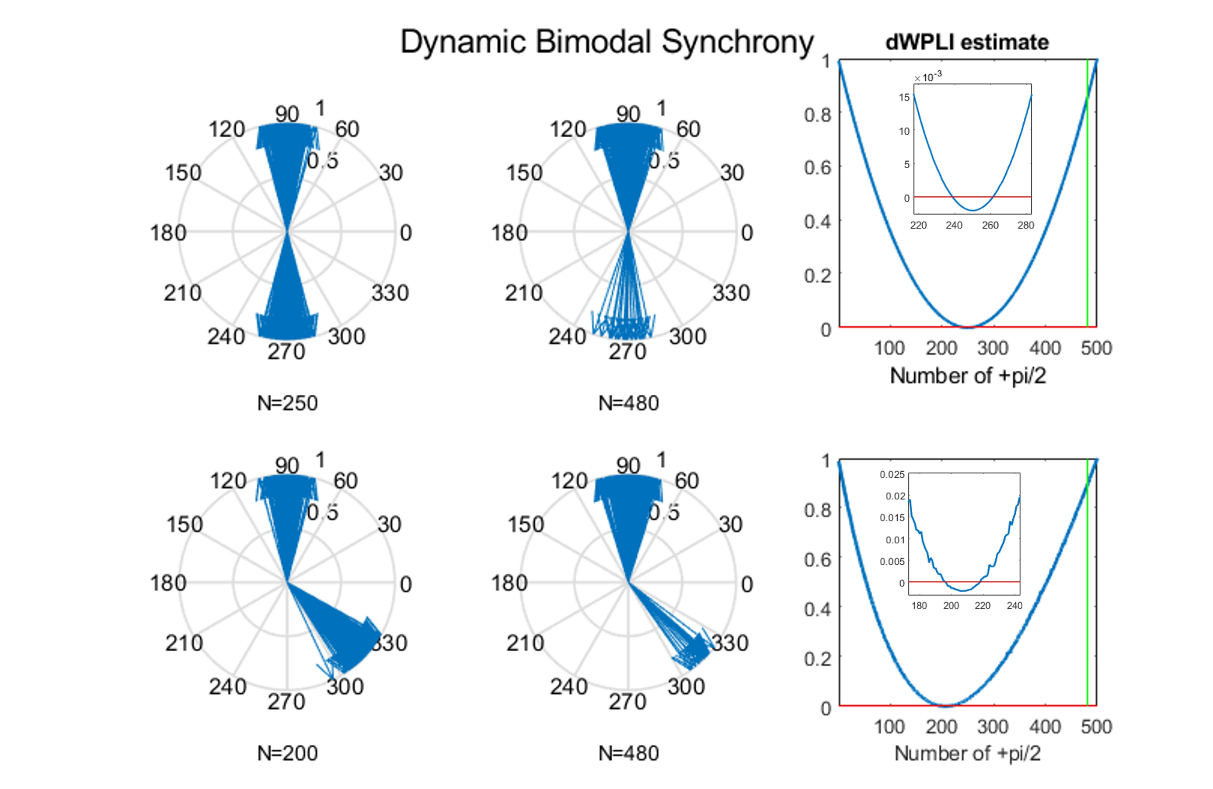


**Figure S3.** Two cases of opposite-sign dynamic bimodal distributions synchrony. Top row: 500 simulated phase difference angles are located anti-phase around $\frac{\pi}{2}$ and $-\frac{\pi}{2}$. DWPLI estimates form a symmetric convex as sample size shifts between the two groups. Bottom row: simulated phase difference angles are grouped around $\frac{\pi}{2}$ and $-\frac{\pi}{4}$. Green vertical line marks the dWPLI estimate at N=480.

4

Figure S3. Left: Randomly generated phase difference angles plotted on unit circle. Right: Simulation shows dWPLI converges to zero as sample size increases.


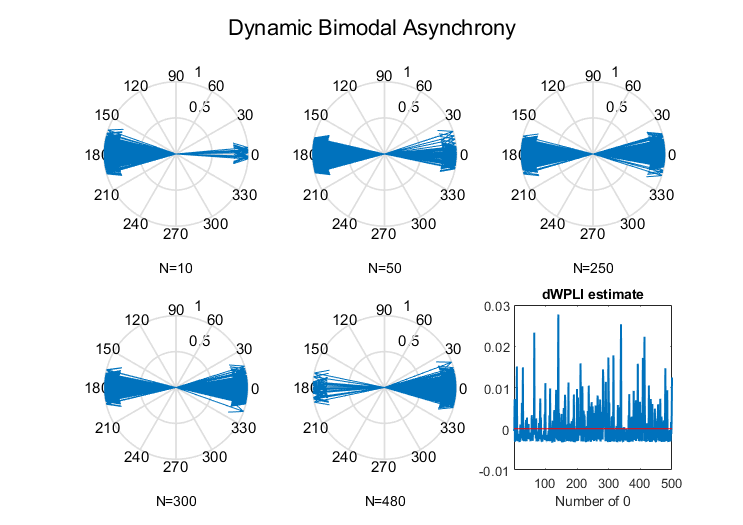


**Figure S4.** Simulations of bimodal extremes on volume conduction angles (0 and 180 degrees). No matter the sample sizes change between groups, dWPLI estimates are close to zero and negatively biased.

1. Grinsted A, Moore JC, Jevrejeva S. Application of the cross wavelet transform and wavelet coherence to geophysical time series. Nonlin Processes Geophys. 2004;11(5/6):561-6.
