# Supplementary material for: Altered frontal connectivity as a mechanism for executive function deficits in fragile X syndrome
Source: Mol Autism. 2022 Dec 9;13:47. doi: 10.1186/s13229-022-00527-0 (PMC9733336; doi:10.1186/s13229-022-00527-0)
Supplement: Supplementary file 3 — Additional file 3. Post hoc pair comparisons of group from lme models for gamma and alpha band connectivity. [file 13229_2022_527_MOESM3_ESM.docx]

Post hoc pair comparisons of group from lme models

**Table S1. Gamma band left frontal region pairwise comparisons of group.**

| Contrast | sex | connection | estimate | P-value | FDR adjusted P | Estimate  After IQ accounted | P-value  After IQ accounted | FDR adjusted P  After IQ accounted |
| --- | --- | --- | --- | --- | --- | --- | --- | --- |
| FXS-TDC | male | cMFG-pOPER | 0.1635 | 0.0036** | 0.0190* | 0.1450 | 0.0772 | 0.2206 |
| FXS-TDC | male | cMFG-pTRI | 0.0660 | 0.2366 | 0.4485 | 0.1258 | 0.1234 | 0.2244 |
| FXS-TDC | male | cMFG-rMFG | 0.0404 | 0.4676 | 0.6235 | 0.0854 | 0.2940 | 0.4200 |
| FXS-TDC | male | cMFG-sFG | 0.0049 | 0.9289 | 0.9289 | 0.0870 | 0.2850 | 0.4200 |
| FXS-TDC | male | pOPER-pTRI | 0.0503 | 0.3716 | 0.5490 | 0.0813 | 0.3208 | 0.4277 |
| FXS-TDC | male | pOPER-rMFG | -0.0161 | 0.7729 | 0.8261 | 0.0553 | 0.4975 | 0.5237 |
| FXS-TDC | male | pOPER-sFG | 0.0483 | 0.3843 | 0.5490 | 0.0632 | 0.4369 | 0.4941 |
| FXS-TDC | male | pTRI-rMFG | 0.1683 | 0.0028** | 0.0190* | 0.1698 | 0.0389* | 0.1297 |
| FXS-TDC | male | pTRI-sFG | 0.0342 | 0.5379 | 0.6709 | 0.0653 | 0.4224 | 0.4941 |
| FXS-TDC | male | rMFG-sFG | 0.0940 | 0.0912 | 0.2222 | 0.1275 | 0.1182 | 0.2244 |
| FXS-TDC | female | cMFG-pOPER | -0.0173 | 0.7848 | 0.8261 | -0.0542 | 0.4447 | 0.4941 |
| FXS-TDC | female | cMFG-pTRI | 0.1030 | 0.1000 | 0.2222 | 0.1133 | 0.1058 | 0.2244 |
| FXS-TDC | female | cMFG-rMFG | 0.0355 | 0.5703 | 0.6709 | 0.0228 | 0.7441 | 0.7441 |
| FXS-TDC | female | cMFG-sFG | 0.1809 | 0.0038** | 0.0190* | 0.1979 | 0.0048** | 0.0320* |
| FXS-TDC | female | pOPER-pTRI | 0.1680 | 0.0085** | 0.0283* | 0.1728 | 0.0157* | 0.0744 |
| FXS-TDC | female | pOPER-rMFG | 0.1653 | 0.0084** | 0.0283* | 0.1655 | 0.0186* | 0.0744 |
| FXS-TDC | female | pOPER-sFG | 0.1432 | 0.0224* | 0.0640 | 0.2143 | 0.0024** | 0.0240* |
| FXS-TDC | female | pTRI-rMFG | 0.2166 | 0.0006*** | 0.0120* | 0.2780 | 0.0001*** | 0.0020** |
| FXS-TDC | female | pTRI-sFG | 0.0724 | 0.2467 | 0.4485 | 0.1183 | 0.0914 | 0.2244 |
| FXS-TDC | female | rMFG-sFG | 0.0621 | 0.3189 | 0.5315 | 0.0777 | 0.2653 | 0.4200 |

**Table S2. Gamma band right frontal region pairwise comparisons of group.**

| Contrast | sex | connection | estimate | P-value | FDR adjusted P | Estimate after IQ adjusted | P-value after IQ adjusted | FDR adjusted P after IQ adjusted |
| --- | --- | --- | --- | --- | --- | --- | --- | --- |
| FXS-TDC | male | cMFG-pOPER | 0.0164 | 0.7935 | 0.7935 | -0.0274 | 0.7718 | 0.8124 |
| FXS-TDC | male | cMFG-pTRI | 0.0917 | 0.1350 | 0.1588 | 0.0220 | 0.8144 | 0.8144 |
| FXS-TDC | male | cMFG-rMFG | 0.1963 | 0.0014** | 0.0040** | 0.1295 | 0.1667 | 0.3056 |
| FXS-TDC | male | cMFG-sFG | 0.1267 | 0.0392* | 0.0603 | 0.0633 | 0.4988 | 0.6651 |
| FXS-TDC | male | pOPER-pTRI | 0.1983 | 0.0013** | 0.0040** | 0.1293 | 0.1681 | 0.3056 |
| FXS-TDC | male | pOPER-rMFG | 0.2102 | 0.0007*** | 0.0040** | 0.1216 | 0.1945 | 0.3242 |
| FXS-TDC | male | pOPER-sFG | 0.0947 | 0.1222 | 0.1528 | -0.0348 | 0.7095 | 0.7883 |
| FXS-TDC | male | pTRI-rMFG | 0.1162 | 0.0585 | 0.0780 | 0.0647 | 0.4892 | 0.6651 |
| FXS-TDC | male | pTRI-sFG | 0.1480 | 0.0161* | 0.0293* | 0.0508 | 0.5862 | 0.6896 |
| FXS-TDC | male | rMFG-sFG | 0.1979 | 0.0013** | 0.0040** | 0.1659 | 0.0771 | 0.2108 |
| FXS-TDC | female | cMFG-pOPER | 0.1409 | 0.0454* | 0.0649 | 0.1386 | 0.0843 | 0.2108 |
| FXS-TDC | female | cMFG-pTRI | 0.1671 | 0.0158* | 0.0293* | 0.1846 | 0.0196* | 0.0784 |
| FXS-TDC | female | cMFG-rMFG | 0.0694 | 0.3123 | 0.3287 | 0.0437 | 0.5774 | 0.6896 |
| FXS-TDC | female | cMFG-sFG | 0.2740 | 0.0001*** | 0.0020** | 0.2342 | 0.0031** | 0.0310* |
| FXS-TDC | female | pOPER-pTRI | 0.2383 | 0.0006*** | 0.0040** | 0.2376 | 0.0028** | 0.0310* |
| FXS-TDC | female | pOPER-rMFG | 0.1527 | 0.0274* | 0.0457* | 0.1258 | 0.1117 | 0.2482 |
| FXS-TDC | female | pOPER-sFG | 0.2315 | 0.0009*** | 0.0040** | 0.1888 | 0.0176* | 0.0784 |
| FXS-TDC | female | pTRI-rMFG | 0.2146 | 0.0020** | 0.0044** | 0.2108 | 0.0079** | 0.0527 |
| FXS-TDC | female | pTRI-sFG | 0.2186 | 0.0016** | 0.0040** | 0.1704 | 0.0315* | 0.1050 |
| FXS-TDC | female | rMFG-sFG | 0.0955 | 0.1648 | 0.1831 | 0.0664 | 0.3980 | 0.6123 |

***Table S3. Gamma band left prefrontal region pairwise comparisons of group.***

| Contrast | sex | connection | estimate | P-value | FDR adjusted P | Estimate after IQ adjusted | P-value after IQ adjusted | FDR adjusted P after IQ adjusted |
| --- | --- | --- | --- | --- | --- | --- | --- | --- |
| FXS-TDC | male | FP-LOF | -0.0361 | 0.6566 | 0.6566 | 0.1893 | 0.0889 | 0.0889 |
| FXS-TDC | male | FP-MOF | 0.1135 | 0.1636 | 0.2454 | 0.3552 | 0.0016** | 0.0031** |
| FXS-TDC | male | FP-pORB | -0.0458 | 0.5737 | 0.6259 | 0.2146 | 0.0545 | 0.0595 |
| FXS-TDC | male | LOF-MOF | 0.2118 | 0.0095** | 0.0380* | 0.3708 | 0.0010** | 0.0024** |
| FXS-TDC | male | LOF-pORB | 0.1166 | 0.1521 | 0.2454 | 0.2898 | 0.0096** | 0.0143* |
| FXS-TDC | male | MOF-pORB | 0.1816 | 0.0261* | 0.0783 | 0.3815 | 0.0007*** | 0.0021** |
| FXS-TDC | female | FP-LOF | 0.2622 | 0.0044** | 0.0264* | 0.3971 | <.0001*** | <.0001*** |
| FXS-TDC | female | FP-MOF | 0.0919 | 0.3149 | 0.3779 | 0.2124 | 0.0233* | 0.0280* |
| FXS-TDC | female | FP-pORB | 0.3000 | 0.0011** | 0.0132* | 0.4593 | <.0001*** | <.0001*** |
| FXS-TDC | female | LOF-MOF | 0.1056 | 0.2474 | 0.3299 | 0.2388 | 0.0107* | 0.0143* |
| FXS-TDC | female | LOF-pORB | 0.1966 | 0.0333* | 0.0799 | 0.3238 | 0.0007*** | 0.0021** |
| FXS-TDC | female | MOF-pORB | 0.1821 | 0.0477* | 0.0954 | 0.2940 | 0.0018** | 0.0031** |

***Table S4.1. Gamma band right prefrontal region pairwise comparisons of group from 3-way interaction group:sex:frequency.***

| Contrast | sex | Frequency | estimate | P-value | FDR adjusted P | Estimate after IQ adjusted | P-value after IQ adjusted | FDR adjusted P after IQ adjusted |
| --- | --- | --- | --- | --- | --- | --- | --- | --- |
| FXS-TDC | male | 30Hz | 0.0585 | 0.0464* | 0.0928 | 0.1155 | 0.0149* | 0.0298* |
| FXS-TDC | male | 35Hz | 0.1164 | 0.0001*** | 0.0012** | 0.1612 | 0.0008*** | 0.0096** |
| FXS-TDC | male | 40Hz | 0.0893 | 0.0025** | 0.0150* | 0.1222 | 0.0101* | 0.0242* |
| FXS-TDC | male | 45Hz | 0.0551 | 0.0601 | 0.1030 | 0.0899 | 0.0572 | 0.0686 |
| FXS-TDC | male | 50Hz | 0.0425 | 0.1462 | 0.1595 | 0.0842 | 0.0744 | 0.0812 |
| FXS-TDC | male | 55Hz | 0.0516 | 0.0784 | 0.1169 | 0.1025 | 0.0306* | 0.0459* |
| FXS-TDC | female | 30Hz | 0.0518 | 0.1154 | 0.1385 | 0.0808 | 0.0359* | 0.0479* |
| FXS-TDC | female | 35Hz | 0.0562 | 0.0877 | 0.1169 | 0.0914 | 0.0178* | 0.0305* |
| FXS-TDC | female | 40Hz | 0.0197 | 0.5479 | 0.5479 | 0.0633 | 0.0989 | 0.0989 |
| FXS-TDC | female | 45Hz | 0.0758 | 0.0214* | 0.0514 | 0.1054 | 0.0064** | 0.0192* |
| FXS-TDC | female | 50Hz | 0.0780 | 0.0182* | 0.0514 | 0.1075 | 0.0054** | 0.0192* |
| FXS-TDC | female | 55Hz | 0.0841 | 0.0110* | 0.0440* | 0.1228 | 0.0016** | 0.0096** |

***Table S4.2. Gamma band right prefrontal region pairwise comparisons of group from 2-way interaction group:connection.***

| contrast | connection | estimate | P-value | FDR adjusted P | Estimate after IQ adjusted | P-value after IQ adjusted | FDR adjusted P after IQ adjusted |
| --- | --- | --- | --- | --- | --- | --- | --- |
| FXS-TDC | FP-LOF | 0.0554 | 0.0123* | 0.0185* | 0.0894 | 0.0117* | 0.0142* |
| FXS-TDC | FP-MOF | 0.0390 | 0.0762 | 0.0836 | 0.0789 | 0.0253* | 0.0253* |
| FXS-TDC | FP-pORB | 0.0381 | 0.0836 | 0.0836 | 0.0892 | 0.0118* | 0.0142* |
| FXS-TDC | LOF-MOF | 0.0923 | <.0001**** | <0.0003*** | 0.1436 | 0.0001*** | 0.0006*** |
| FXS-TDC | LOF-pORB | 0.0997 | <.0001**** | <0.0003*** | 0.1296 | 0.0003*** | 0.0009*** |
| FXS-TDC | MOF-pORB | 0.0651 | 0.0032** | 0.0064** | 0.0928 | 0.0088** | 0.0142* |

***Table S5. Gamma band cross hemisphere connections pairwise comparisons of group.***

| contrast | sex | connection | estimate | P-value | FDR adjusted P | Estimate after IQ adjusted | P-value after IQ adjusted | FDR adjusted P after IQ adjusted |
| --- | --- | --- | --- | --- | --- | --- | --- | --- |
| FXS-TDC | male | cMFG | -0.0010 | 0.9770 | 0.9770 | 0.0411 | 0.4310 | 0.4393 |
| FXS-TDC | male | FP | 0.0837 | 0.0170* | 0.0306* | 0.1650 | 0.0018** | 0.0046** |
| FXS-TDC | male | LOF | 0.0889 | 0.0112* | 0.0288* | 0.1361 | 0.0097** | 0.0175* |
| FXS-TDC | male | MOF | 0.1291 | 0.0002*** | 0.0036** | 0.1535 | 0.0036** | 0.0081** |
| FXS-TDC | male | pOPER | 0.0594 | 0.0895 | 0.1239 | 0.0916 | 0.0804 | 0.1034 |
| FXS-TDC | male | pORB | 0.0638 | 0.0687 | 0.1031 | 0.1148 | 0.0289* | 0.0434* |
| FXS-TDC | male | pTRI | 0.1106 | 0.0016** | 0.0058** | 0.1831 | 0.0005*** | 0.0040** |
| FXS-TDC | male | rMFG | 0.0864 | 0.0138* | 0.0306* | 0.1371 | 0.0092** | 0.0175* |
| FXS-TDC | male | sFG | 0.0353 | 0.3134 | 0.3761 | 0.0835 | 0.1107 | 0.1328 |
| FXS-TDC | female | cMFG | 0.1272 | 0.0013** | 0.0058** | 0.1554 | 0.0007*** | 0.0040** |
| FXS-TDC | female | FP | 0.0304 | 0.4415 | 0.4967 | 0.0548 | 0.2289 | 0.2575 |
| FXS-TDC | female | LOF | 0.0951 | 0.0163* | 0.0306* | 0.1509 | 0.0010** | 0.0040** |
| FXS-TDC | female | MOF | 0.0764 | 0.0526 | 0.0861 | 0.1157 | 0.0111* | 0.0182* |
| FXS-TDC | female | pOPER | 0.1162 | 0.0032** | 0.0096** | 0.1447 | 0.0015** | 0.0045** |
| FXS-TDC | female | pORB | 0.0545 | 0.1692 | 0.2175 | 0.0838 | 0.0664 | 0.0919 |
| FXS-TDC | female | pTRI | 0.1288 | 0.0011** | 0.0058** | 0.1491 | 0.0011** | 0.0040** |
| FXS-TDC | female | rMFG | 0.1394 | 0.0004*** | 0.0036** | 0.1872 | <.0001*** | <.0001*** |
| FXS-TDC | female | sFG | 0.0176 | 0.6547 | 0.6932 | 0.0350 | 0.4393 | 0.4393 |

***Table S6: Alpha band right frontal region pairwise comparisons of group.***

| contrast | sex | connection | estimate | P-value | FDR adjusted P | Estimate after IQ adjusted | P-value after IQ adjusted | FDR adjusted P after IQ adjusted |
| --- | --- | --- | --- | --- | --- | --- | --- | --- |
| FXS-TDC | male | cMFG-pOPER | -0.0932 | 0.0162* | 0.0540 | -0.0919 | 0.1430 | 0.3178 |
| FXS-TDC | male | cMFG-pTRI | 0.0468 | 0.2231 | 0.2975 | 0.0645 | 0.3023 | 0.4319 |
| FXS-TDC | male | cMFG-rMFG | -0.0778 | 0.0434* | 0.0964 | -0.0952 | 0.1288 | 0.3178 |
| FXS-TDC | male | cMFG-sFG | -0.0235 | 0.5407 | 0.6008 | -0.0656 | 0.2945 | 0.4319 |
| FXS-TDC | male | pOPER-pTRI | 0.0308 | 0.4231 | 0.4978 | 0.0352 | 0.5728 | 0.6739 |
| FXS-TDC | male | pOPER-rMFG | -0.0056 | 0.8829 | 0.8829 | -0.0017 | 0.9780 | 0.9780 |
| FXS-TDC | male | pOPER-sFG | -0.1173 | 0.0025** | 0.0440* | -0.1646 | 0.0091** | 0.1820 |
| FXS-TDC | male | pTRI-rMFG | -0.0595 | 0.1215 | 0.1773 | -0.0248 | 0.6913 | 0.7681 |
| FXS-TDC | male | pTRI-sFG | -0.1060 | 0.0061** | 0.0440* | -0.1118 | 0.0747 | 0.2490 |
| FXS-TDC | male | rMFG-sFG | -0.0980 | 0.0110* | 0.0440* | -0.0794 | 0.2040 | 0.4080 |
| FXS-TDC | female | cMFG-pOPER | -0.0083 | 0.8481 | 0.8829 | -0.0130 | 0.7991 | 0.8412 |
| FXS-TDC | female | cMFG-pTRI | -0.1109 | 0.0105* | 0.0440* | -0.1157 | 0.0244* | 0.2440 |
| FXS-TDC | female | cMFG-rMFG | -0.0977 | 0.0243* | 0.0663 | -0.1032 | 0.0449* | 0.2490 |
| FXS-TDC | female | cMFG-sFG | -0.0732 | 0.0917 | 0.1528 | -0.0861 | 0.0953 | 0.2723 |
| FXS-TDC | female | pOPER-pTRI | -0.0428 | 0.3203 | 0.4004 | -0.0555 | 0.2780 | 0.4319 |
| FXS-TDC | female | pOPER-rMFG | -0.0777 | 0.0723 | 0.1315 | -0.0582 | 0.2555 | 0.4319 |
| FXS-TDC | female | pOPER-sFG | -0.0963 | 0.0265* | 0.0663 | -0.0924 | 0.0726 | 0.2490 |
| FXS-TDC | female | pTRI-rMFG | -0.0830 | 0.0547 | 0.1094 | -0.0393 | 0.4421 | 0.5526 |
| FXS-TDC | female | pTRI-sFG | -0.1140 | 0.0086** | 0.0440* | -0.1004 | 0.0506 | 0.2490 |
| FXS-TDC | female | rMFG-sFG | -0.0665 | 0.1241 | 0.1773 | -0.0465 | 0.3637 | 0.4849 |

***Table S7. Alpha band right prefrontal region pairwise comparisons of group.***

| contrast | connection | estimate | P-value | FDR adjusted P | Estimate after IQ adjusted | P-value after IQ adjusted | FDR adjusted P after IQ adjusted |
| --- | --- | --- | --- | --- | --- | --- | --- |
| FXS-TDC | FP-LOF | -0.0680 | 0.0622 | 0.1244 | -0.0554 | 0.3406 | 0.5109 |
| FXS-TDC | FP-MOF | -0.0117 | 0.7470 | 0.7470 | 0.0116 | 0.8419 | 0.8586 |
| FXS-TDC | FP-pORB | -0.0199 | 0.5839 | 0.7007 | 0.0103 | 0.8586 | 0.8586 |
| FXS-TDC | LOF-MOF | -0.0602 | 0.0987 | 0.1481 | -0.0600 | 0.3020 | 0.5109 |
| FXS-TDC | LOF-pORB | -0.1777 | <.0001**** | <0.0006*** | -0.1604 | 0.0064** | 0.0384* |
| FXS-TDC | MOF-pORB | -0.1384 | 0.0002*** | 0.0006*** | -0.1297 | 0.0268* | 0.0804 |

Post hoc pair comparisons of sex from lme models

***Table S8. Alpha band cross hemisphere connections pairwise comparisons of sex.***

| contrast | connection | estimate | P-value | FDR adjusted P | Estimate after IQ adjusted | P-value after IQ adjusted | FDR adjusted P after IQ adjusted |
| --- | --- | --- | --- | --- | --- | --- | --- |
| male-female | cMFG | -0.1397 | 0.1474 | 0.4422 | -0.1536 | 0.1631 | 0.4893 |
| male-female | FP | 0.1901 | 0.0474* | 0.2133 | 0.2175 | 0.0484* | 0.2178 |
| male-female | LOF | -0.0258 | 0.7862 | 0.9685 | -0.0098 | 0.9287 | 0.9287 |
| male-female | MOF | 0.0323 | 0.7355 | 0.9685 | 0.0304 | 0.7815 | 0.9287 |
| male-female | pOPER | -0.0009 | 0.9922 | 0.9922 | 0.0231 | 0.8330 | 0.9287 |
| male-female | pORB | -0.0167 | 0.8609 | 0.9685 | 0.0173 | 0.8744 | 0.9287 |
| male-female | pTRI | -0.0827 | 0.3855 | 0.8674 | -0.0781 | 0.4746 | 0.9287 |
| male-female | rMFG | -0.3564 | 0.0003*** | 0.0027** | -0.3844 | 0.0006*** | 0.0054** |
| male-female | sFG | 0.0445 | 0.6402 | 0.9685 | 0.0265 | 0.8087 | 0.9287 |

Post hoc pair comparisons of stimulant from lme models

***Table S9: Alpha band right frontal region pairwise comparisons of stimulant.***

| contrast | sex | connection | estimate | P-value | FDR adjusted P |
| --- | --- | --- | --- | --- | --- |
| on-off | male | cMFG-pOPER | 0.1057 | 0.0516 | 0.1720 |
| on-off | male | cMFG-pTRI | 0.0939 | 0.0812 | 0.1804 |
| on-off | male | cMFG-rMFG | 0.0552 | 0.3012 | 0.4303 |
| on-off | male | cMFG-sFG | 0.1141 | 0.0354* | 0.1416 |
| on-off | male | pOPER-pTRI | 0.0452 | 0.3969 | 0.4961 |
| on-off | male | pOPER-rMFG | 0.0178 | 0.7377 | 0.7377 |
| on-off | male | pOPER-sFG | 0.0328 | 0.5397 | 0.5696 |
| on-off | male | pTRI-rMFG | 0.0772 | 0.1484 | 0.2508 |
| on-off | male | pTRI-sFG | 0.0752 | 0.1593 | 0.2508 |
| on-off | male | rMFG-sFG | 0.1569 | 0.0037** | 0.0440* |
| on-off | female | cMFG-pOPER | -0.0796 | 0.1597 | 0.2508 |
| on-off | female | cMFG-pTRI | -0.1361 | 0.0169* | 0.0845 |
| on-off | female | cMFG-rMFG | -0.0792 | 0.1630 | 0.2508 |
| on-off | female | cMFG-sFG | -0.0375 | 0.5061 | 0.5696 |
| on-off | female | pOPER-pTRI | -0.1633 | 0.0044* | 0.0440* |
| on-off | female | pOPER-rMFG | -0.1033 | 0.0685 | 0.1804 |
| on-off | female | pOPER-sFG | -0.0345 | 0.5411 | 0.5696 |
| on-off | female | pTRI-rMFG | -0.1423 | 0.0126* | 0.0840 |
| on-off | female | pTRI-sFG | -0.0993 | 0.0797 | 0.1804 |
| on-off | female | rMFG-sFG | -0.0553 | 0.3279 | 0.4372 |

***Table S10. Alpha band right prefrontal region pairwise comparisons of stimulant.***

| contrast | sex | connection | estimate | P-value | FDR adjusted P |
| --- | --- | --- | --- | --- | --- |
| on-off | male | FP-LOF | 0.0040 | 0.9505 | 0.9948 |
| on-off | male | FP-MOF | -0.0577 | 0.3777 | 0.5036 |
| on-off | male | FP-pORB | 0.0636 | 0.3310 | 0.4965 |
| on-off | male | LOF-MOF | 0.1904 | 0.0043** | 0.0232* |
| on-off | male | LOF-pORB | 0.1123 | 0.0871 | 0.2613 |
| on-off | male | MOF-pORB | -0.0004 | 0.9948 | 0.9948 |
| on-off | female | FP-LOF | -0.0685 | 0.3237 | 0.4965 |
| on-off | female | FP-MOF | -0.0676 | 0.3298 | 0.4965 |
| on-off | female | FP-pORB | -0.0952 | 0.1703 | 0.4087 |
| on-off | female | LOF-MOF | -0.0537 | 0.4383 | 0.5260 |
| on-off | female | LOF-pORB | -0.1940 | 0.0058** | 0.0232* |
| on-off | female | MOF-pORB | -0.2568 | 0.0003*** | 0.0036** |

***Table S11. Alpha band cross hemisphere connections pairwise comparisons of stimulant.***

| contrast | sex | connection | estimate | P-value | FDR adjusted P |
| --- | --- | --- | --- | --- | --- |
| on-off | male | cMFG | 0.2413 | 0.0759 | 0.2277 |
| on-off | male | FP | 0.1728 | 0.1996 | 0.4314 |
| on-off | male | LOF | 0.0746 | 0.5789 | 0.6947 |
| on-off | male | MOF | 0.0649 | 0.6331 | 0.7122 |
| on-off | male | pOPER | -0.0260 | 0.8464 | 0.8464 |
| on-off | male | pORB | 0.1307 | 0.3306 | 0.5410 |
| on-off | male | pTRI | 0.5248 | 0.0001*** | 0.0018** |
| on-off | male | rMFG | 0.1665 | 0.2157 | 0.4314 |
| on-off | male | sFG | 0.1368 | 0.3094 | 0.5410 |
| on-off | female | cMFG | -0.2904 | 0.0443* | 0.1994 |
| on-off | female | FP | -0.1234 | 0.3860 | 0.5790 |
| on-off | female | LOF | -0.2724 | 0.0568 | 0.2045 |
| on-off | female | MOF | -0.3414 | 0.0174* | 0.1566 |
| on-off | female | pOPER | -0.1969 | 0.1682 | 0.4314 |
| on-off | female | pORB | -0.3093 | 0.0312 | 0.1872 |
| on-off | female | pTRI | -0.0965 | 0.4974 | 0.6395 |
| on-off | female | rMFG | -0.0415 | 0.7703 | 0.8156 |
| on-off | female | sFG | -0.1020 | 0.4736 | 0.6395 |

***Table S12. Alpha band cross hemisphere connections pairwise comparisons of sex without 10 mosaic males.***

| contrast | connection | estimate | P-value | FDR adjusted P | Estimate after IQ adjusted | P-value after IQ adjusted | FDR adjusted P after IQ adjusted |
| --- | --- | --- | --- | --- | --- | --- | --- |
| male-female | cMFG | -0.2319 | 0.0303 | 0.1364 | -0.2343 | 0.0731 | 0.2193 |
| male-female | FP | 0.2088 | 0.0491 | 0.1473 | 0.2730 | 0.0372 | 0.1674 |
| male-female | LOF | 0.0494 | 0.6392 | 0.7191 | 0.1022 | 0.4303 | 0.6075 |
| male-female | MOF | 0.0587 | 0.5792 | 0.7191 | 0.0797 | 0.5400 | 0.6075 |
| male-female | pOPER | 0.0668 | 0.5262 | 0.7191 | 0.1303 | 0.3053 | 0.5675 |
| male-female | pORB | -0.0345 | 0.7427 | 0.7427 | 0.0359 | 0.7815 | 0.7815 |
| male-female | pTRI | -0.1108 | 0.2925 | 0.5265 | -0.0850 | 0.5108 | 0.6075 |
| male-female | rMFG | -0.2975 | 0.0053** | 0.0477* | -0.3115 | 0.0177 | 0.1593 |
| male-female | sFG | 0.1782 | 0.0917 | 0.2063 | 0.1862 | 0.1518 | 0.3416 |
