# Supplementary material for: Altered frontal connectivity as a mechanism for executive function deficits in fragile X syndrome
Source: Mol Autism. 2022 Dec 9;13:47. doi: 10.1186/s13229-022-00527-0 (PMC9733336; doi:10.1186/s13229-022-00527-0)
Supplement: Supplementary file 4 — Additional file 4. Summary of KiTAP performance of individuals with FXS taking stimulants (FXS+stimulant), those not taking stimulants (FXS-stimulant), and typically-developing controls (TDC). [file 13229_2022_527_MOESM4_ESM.docx]

**Table S13. Summary of KiTAP performance of individuals with FXS taking stimulants (FXS+stimulant) and those not taking stimulants (FXS-stimulant)**

|  |  | **FXS+stimulant** | **FXS-stimulant** |
| --- | --- | --- | --- |
| **Alertness** | RT median | 538.43 (293) | 664.62 (409) |
|  | RT SD | 257.81 (271) | 346.18 (381) |
| **Distractibility** | RT median | 551.68 (226) | 569.16 (232) |
|  | Errors | 15.58 (12) | 16.11 (13) |
| **Flexibility** | RT median | 1136.32 (473) | 1193.67 (759) |
|  | Errors | 10.53 (4) | 8.4 (5) |
| **Go/NoGo** | RT median | 510.10 (198) | 483.69 (99) |
|  | Errors | 3.7 (4) | 4.94 (6) |

RT=reaction time, SD=standard deviation; * p<.05, ** p<.01, *** p<.001

**Table S14. Summary of KiTAP performance of FXS+stimulant and TDC**

|  |  | **FXS+stimulant** | **TDC** |
| --- | --- | --- | --- |
| **Alertness** | RT median | 538.43 (293)*** | 344.85 (103) |
|  | RT SD | 257.81 (271)*** | 61.70 (44) |
| **Distractibility** | RT median | 551.68 (226)*** | 464.10 (766) |
|  | Errors | 15.58 (12)*** | 5.34 (7) |
| **Flexibility** | RT median | 1136.32 (473)*** | 699.79 (262) |
|  | Errors | 10.53 (4)*** | 0.83 (1) |
| **Go/NoGo** | RT median | 510.10 (198)** | 444.39 (82) |
|  | Errors | 3.7 (4)*** | 0.8 (2) |

RT=reaction time, SD=standard deviation; * p<.05, ** p<.01, *** p<.001

**Table S15. Summary of KiTAP performance of FXS-stimulant and TDC**

|  |  | **FXS-stimulant** | **TDC** |
| --- | --- | --- | --- |
| **Alertness** | RT median | 664.62 (409)*** | 344.85 (103) |
|  | RT SD | 346.18 (381)*** | 61.70 (44) |
| **Distractibility** | RT median | 569.16 (232)** | 464.10 (766) |
|  | Errors | 16.11 (13)*** | 5.34 (7) |
| **Flexibility** | RT median | 1193.67 (759)*** | 699.79 (262) |
|  | Errors | 8.4 (5)*** | 0.83 (1) |
| **Go/NoGo** | RT median | 483.69 (99)** | 444.39 (82) |
|  | Errors | 4.94 (6)*** | 0.8 (2) |

RT=reaction time, SD=standard deviation; * p<.05, ** p<.01, *** p<.001
