# Supplementary material for: Altered frontal connectivity as a mechanism for executive function deficits in fragile X syndrome
Source: Mol Autism. 2022 Dec 9;13:47. doi: 10.1186/s13229-022-00527-0 (PMC9733336; doi:10.1186/s13229-022-00527-0)
Supplement: Supplementary file 5 — Additional file 5. Spearman’s Partial Correlations (Accounting for Nonverbal IQ and Age) Gamma Connectivity Strength and Executive Function as well as Alpha Connectivity Strength and Executive Function in FXS. [file 13229_2022_527_MOESM5_ESM.docx]

**Table S16.** Spearman’s Partial Correlations (Accounting for Nonverbal IQ and Age) between Gamma Connectivity Strength and Executive Function in FXS

|  |  | | | | | | | | | |
| --- | --- | --- | --- | --- | --- | --- | --- | --- | --- | --- |
|  | **Cross Hemi** | | **Left Frontal** | | **Left Prefrontal** | | **Right Frontal** | | **Right Prefrontal** | |
| Variable | rho | p | rho | p | rho | p | rho | p | rho | p |
| **Alert Median Time** |  |  |  |  |  |  |  |  |  |  |
| FXS Males | 0.163 | 0.400 | 0.096 | 0.622 | 0.151 | 0.435 | 0.158 | 0.413 | 0.339 | 0.072 |
| FXS Females | -0.195 | 0.340 | -0.072 | 0.727 | -0.028 | 0.894 | -0.092 | 0.657 | -0.065 | 0.753 |
| FXS All | 0.066 | 0.628 | -0.007 | 0.958 | 0.031 | 0.817 | 0.040 | 0.768 | 0.159 | 0.237 |
| **Alert Standard Deviation** |  |  |  |  |  |  |  |  |  |  |
| FXS Males | 0.017 | 0.931 | 0.103 | 0.594 | -0.044 | 0.822 | 0.081 | 0.676 | 0.166 | 0.390 |
| FXS Females | -0.009 | 0.967 | 0.161 | 0.432 | 0.085 | 0.681 | -0.034 | 0.870 | -0.065 | 0.752 |
| FXS All | 0.064 | 0.636 | 0.014 | 0.919 | -0.023 | 0.867 | 0.015 | 0.915 | 0.130 | 0.334 |
| **Distract Errors** |  |  |  |  |  |  |  |  |  |  |
| FXS Males | 0.080 | 0.705 | 0.569 | 0.003** | 0.252 | 0.223 | 0.289 | 0.162 | 0.041 | 0.847 |
| FXS Females | 0.121 | 0.555 | 0.133 | 0.516 | 0.009 | 0.966 | 0.072 | 0.726 | 0.038 | 0.854 |
| FXS All | 0.100 | 0.478 | 0.342 | 0.012* | 0.187 | 0.181 | 0.180 | 0.196 | -0.013 | 0.929 |
| **Distract Median Time** |  |  |  |  |  |  |  |  |  |  |
| FXS Males | 0.181 | 0.387 | -0.069 | 0.744 | -0.215 | 0.301 | -0.068 | 0.745 | 0.244 | 0.240 |
| FXS Females | -0.286 | 0.157 | -0.258 | 0.202 | -0.214 | 0.294 | -0.218 | 0.284 | -0.279 | 0.167 |
| FXS All | -0.095 | 0.500 | -0.174 | 0.212 | -0.218 | 0.117 | -0.115 | 0.413 | 0.004 | 0.980 |

|  | **Cross Hemi** | | **Left Frontal** | | **Left Prefrontal** | | **Right Frontal** | | **Right Prefrontal** | |
| --- | --- | --- | --- | --- | --- | --- | --- | --- | --- | --- |
| **Flex Error** |  |  |  |  |  |  |  |  |  |  |
| FXS Males | 0.132 | 0.557 | -0.019 | 0.933 | 0.262 | 0.240 | 0.106 | 0.638 | 0.077 | 0.732 |
| FXS Females | -0.461 | 0.031* | -0.159 | 0.479 | -0.294 | 0.184 | -0.284 | 0.200 | -0.521 | 0.013* |
| FXS All | -0.089 | 0.558 | -0.087 | 0.567 | 0.028 | 0.855 | -0.041 | 0.789 | -0.204 | 0.173 |
| **Flex Median Time** |  |  |  |  |  |  |  |  |  |  |
| FXS Males | 0.017 | 0.942 | -0.535 | 0.010** | -0.122 | 0.590 | -0.162 | 0.472 | 0.098 | 0.664 |
| FXS Females | -0.213 | 0.342 | 0.146 | 0.517 | -0.023 | 0.919 | 0.058 | 0.799 | 0.056 | 0.805 |
| FXS All | -0.114 | 0.452 | -0.209 | 0.164 | -0.097 | 0.520 | -0.093 | 0.539 | 0.065 | 0.666 |
| **Go/Nogo Error** |  |  |  |  |  |  |  |  |  |  |
| FXS Males | 0.229 | 0.260 | 0.581 | 0.002** | 0.170 | 0.405 | 0.495 | 0.010** | 0.224 | 0.271 |
| FXS Females | 0.374 | 0.072 | 0.393 | 0.057 | 0.003 | 0.987 | 0.120 | 0.577 | 0.042 | 0.847 |
| FXS All | 0.261 | 0.062 | 0.451 | 0.001*** | 0.123 | 0.386 | 0.323 | 0.020* | 0.120 | 0.396 |
| **Go/Nogo Median Time** |  |  |  |  |  |  |  |  |  |  |
| FXS Males | 0.045 | 0.826 | -0.134 | 0.515 | -0.030 | 0.884 | 0.227 | 0.266 | 0.157 | 0.445 |
| FXS Females | -0.256 | 0.227 | -0.119 | 0.581 | -0.087 | 0.687 | -0.255 | 0.229 | -0.144 | 0.503 |
| FXS All | -0.159 | 0.261 | -0.085 | 0.548 | -0.058 | 0.683 | -0.025 | 0.862 | 0.000 | 0.999 |

*p≤0.05 **≤0.01 ***≤0.001

**Table S17.** Spearman’s Partial Correlations (Accounting for Nonverbal IQ and Age) between Alpha Connectivity Strength and Executive Function in FXS

|  | **Right Frontal** | | **Right Prefrontal** | |
| --- | --- | --- | --- | --- |
| Variable | rho | p | rho | p |
| **Alert Median Time** |  |  |  |  |
| FXS Males | -0.240 | 0.209 | -0.332 | 0.078 |
| FXS Females | 0.143 | 0.487 | 0.004 | 0.985 |
| FXS All | -0.106 | 0.433 | -0.099 | 0.462 |
| **Alert Standard Deviation** |  |  |  |  |
| FXS Males | -0.305 | 0.108 | -0.268 | 0.159 |
| FXS Females | -0.089 | 0.666 | 0.072 | 0.728 |
| FXS All | -0.163 | 0.226 | -0.077 | 0.570 |
| **Distract Errors** |  |  |  |  |
| FXS Males | -0.212 | 0.310 | -0.324 | 0.115 |
| FXS Females | -0.312 | 0.121 | -0.198 | 0.332 |
| FXS All | -0.251 | 0.070 | -0.214 | 0.125 |
| **Distract Median Time** |  |  |  |  |
| FXS Males | -0.045 | 0.829 | 0.084 | 0.689 |
| FXS Females | 0.240 | 0.239 | -0.043 | 0.834 |
| FXS All | 0.122 | 0.383 | 0.065 | 0.643 |
| **Flex Error** |  |  |  |  |
| FXS Males | -0.164 | 0.466 | -0.330 | 0.134 |
| FXS Females | -0.059 | 0.796 | -0.429 | 0.047* |
| FXS All | -0.203 | 0.175 | -0.322 | 0.029* |
|  | **Right Frontal** | | **Right Prefrontal** | |
| **Flex Median Time** |  |  |  |  |
| FXS Males | -0.024 | 0.916 | -0.083 | 0.714 |
| FXS Females | -0.206 | 0.357 | -0.243 | 0.275 |
| FXS All | -0.099 | 0.512 | -0.114 | 0.451 |
| **Go/Nogo Error** |  |  |  |  |
| FXS Males | -0.178 | 0.385 | -0.189 | 0.355 |
| FXS Females | -0.371 | 0.075 | -0.052 | 0.808 |
| FXS All | -0.233 | 0.097 | -0.117 | 0.410 |
| **Go/Nogo Median Time** |  |  |  |  |
| FXS Males | -0.347 | 0.083 | -0.215 | 0.292 |
| FXS Females | 0.254 | 0.231 | 0.030 | 0.891 |
| FXS All | -0.034 | 0.810 | -0.062 | 0.664 |

*p≤0.05 **≤0.01 ***≤0.001
